# Palmatine potentiates cefquinome efficacy against multidrug-resistant *Escherichia coli* via sulfur/taurine metabolism and oxidative stress modulation

**DOI:** 10.3389/fmicb.2025.1644399

**Published:** 2025-11-14

**Authors:** Yining Zhang, Yinchao Tong, Pengcheng Li, Leixin Zhu, Hao Zhang, Honglin Xie, Saba Nasir, Wei Li, Mingjin Fang, Juan Wang, Suzhu Qing, Xinglong Wang, Weimin Zhang

**Affiliations:** 1 College of Veterinary Medicine, Northwest A&F University, Yangling, China; 2 Institute of Traditional Chinese Veterinary Medicine, Northwest A&F University, Yangling, China

**Keywords:** natural products, multidrug resistant bacteria, palmatine, cefquinome, *Escherichia coli*

## Abstract

Palmatine, a natural isoquinoline alkaloid derived from *Fibraureae Caulis*, is widely used for its heat-clearing, detoxifying, antibacterial, and anti-inflammatory properties. The emergence of multidrug-resistant *Escherichia coli* poses a critical challenge to the efficacy of β-lactam antibiotics, particularly cephalosporins such as cefquinome. This study demonstrates that palmatine markedly enhances the antibacterial activity of cefquinome through multi-targeted mechanisms, revealing a new pharmacological potential for this compound. Antimicrobial and synergistic activities were assessed using the microbroth dilution method and checkerboard assay. Bacterial morphology was examined using scanning electron microscopy (SEM), while biofilm inhibition was assessed using confocal laser microscopy. Membrane damage and reactive oxygen species (ROS) levels were detected with fluorescent probe dyes. Transcriptome and RT-qPCR analyses were conducted to identify key mechanistic pathways, and the synergistic effect was further validated in a mouse *Escherichia coli* infection model. *In vitro* analyses of 20 tested isolates revealed broad synergistic effects (FICI ≤ 0.5), with cefquinome MICs reduced by 4- and 32-fold. Mechanistic studies revealed that palmatine disrupts membrane integrity, potentiates oxidative stress, and inhibits biofilm formation. Transcriptomic profiling implicated sulfur metabolism as a key pathway, showing that palmatine reversed cefquinome-induced downregulation of sulfur metabolism-related genes. Functional validation confirmed that disruption of taurine uptake in the sulfur metabolic pathway eliminated the synergistic effect. In murine infection models, the combination therapy increased survival by 30%, alleviated diarrhea, and significantly reduced bacterial loads in tissue. This study reveals the novel pharmacological properties of palmatine, identifies metabolic-level reversal regulation as a novel strategy to combat β-lactam resistance, and highlights palmatine as a multi-target adjuvant that enhances cefquinome efficacy against resistant Gram-negative infections.

## Graphical abstract

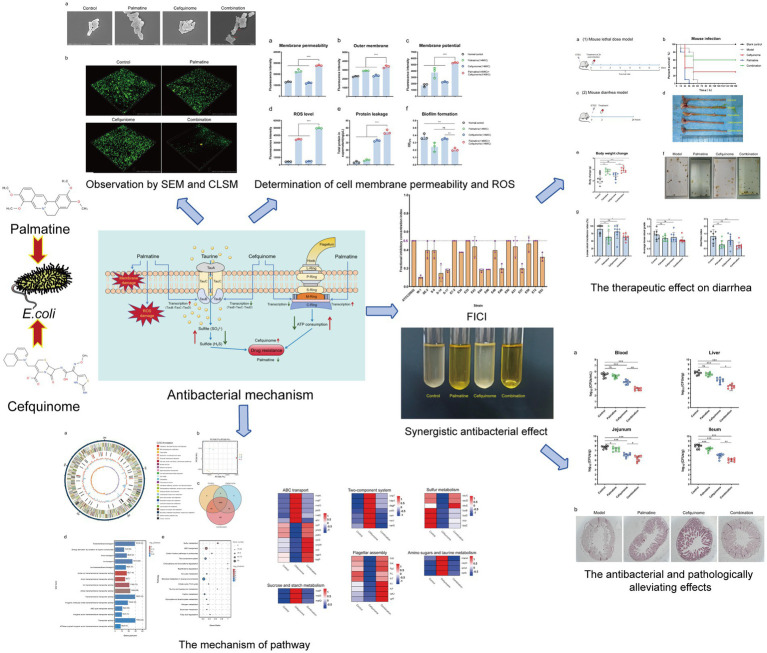

## Highlights

- Broad synergy: All tested strains demonstrate the synergistic effects of palmatine and cefquinome.
- Antibacterial mechanism: Palmatine disrupts membrane integrity, potentiates oxidative stress, and inhibits biofilm formation in *E. coli.*
- Metabolic reversal: Sulfur metabolism is the key pathway to reverse drug resistance.

## 1 Introduction

The emergence of antimicrobial resistance poses a critical public health challenge, undermining our ability to control pathogenic microorganisms and contributing to substantial morbidity and mortality worldwide (Singh and Chibale, 2021; So and Walti, 2022). Recent epidemiological data indicate that drug-resistant bacterial infections account for millions of deaths annually, with *Escherichia coli* being among the most prevalent resistant pathogens (Birlutiu and Birlutiu, 2025).

Despite the urgent demand for new antimicrobial agents, the antibiotic development pipeline remains alarmingly limited, with only a few novel compounds receiving clinical approval in the past two decades, while bacterial resistance continues to escalate at an unprecedented rate (Shariati et al., 2020). The substantial increase in β-lactam resistance in Gram-negative bacteria has been widely reported in medical facilities, public spaces, agricultural settings, and other ecological niches (Goldstein, 2021; Higgins et al., 2023; Navarro et al., 2023; Zheng et al., 2022). Third-generation cephalosporins have been strategic antibiotics in clinical practice for decades (Menkem et al., 2023; Zhao et al., 2021). Recent surveillance data indicate that 68% of isolates were resistant to third-generation cephalosporins, and the case-fatality rate was 45% among patients with bloodstream infections resistant to third-generation cephalosporins (Lester et al., 2022). Equally concerning is the rapid emergence of resistance to fourth-generation cephalosporins (Fu et al., 2020; Wang M. et al., 2020), underscoring the critical need for innovative strategies to combat β-lactam resistance.

Cefquinome, a fourth-generation cephalosporin developed explicitly for veterinary applications (Chin et al., 1992), demonstrates notable stability against β-lactamases and exhibits potent *in vitro* and *in vivo* activity against both Gram-positive and Gram-negative bacteria (Limbert et al., 1991). Nevertheless, the emergence of cefquinome resistance in bacteria derived from animals has increased drastically (Tong et al., 2023a; Tong et al., 2023b), presenting a substantial challenge to its continued clinical utility. Compounding this issue is the role of animal-derived food products as potential vehicles for the dissemination of transferable β-lactamase-encoding genes within human populations (Carattoli, 2008; Livermore et al., 2007).

Given the complex interconnections between antimicrobial resistance in the human, animal, and environmental sectors (McEwen and Collignon, 2018), practical strategies to control resistance from animal sources are essential for protecting human health (Wang Y. et al., 2020). Given the limited options for combating cephalosporin resistance, natural products have emerged as a promising source of agents that modify resistance.

Palmatine, a bioactive isoquinoline alkaloid derived from traditional medicinal plants, has attracted increasing attention for its broad-spectrum pharmacological properties, including antibacterial, anticancer, and anti-inflammatory activities (Long et al., 2019). Palmatine is the main chemical component contained in Fibraureae Caulis, which was first recorded in The Compendium of Materia Medica, and it is also one of its main active ingredients. Palmatine was included in the Chinese Pharmacopeia in 2020, and its effectiveness and safety have been fully guaranteed.

Previous studies have demonstrated its efficacy against various bacterial pathogens (Fan et al., 2020; Iwasa et al., 1998; Li et al., 2015). In the face of escalating antimicrobial resistance, combination therapy has emerged as a pivotal strategy to restore antibiotic efficacy (Liu et al., 2021; Tyers and Wright, 2019).

In this study, we report that palmatine broadly enhances the antibacterial activity of cefquinome against clinically isolated *Escherichia coli* by regulating sulfur/taurine metabolism and oxidative stress. Palmatine improves survival rates and significantly reduces diarrhea symptoms in a murine model of Enterotoxigenic *Escherichia coli* (ETEC) infection. These findings position palmatine as a multi-target adjuvant capable of overcoming cefquinome resistance, offering a promising strategy for combating multidrug-resistant Gram-negative infections.

## 2 Materials and methods

### 2.1 Chemicals and bacterial strains

Palmatine and cefquinome sulfate (both 97% purity, Macklin/Yuanye) were dissolved in sterile hot water. Our laboratory collection provided an *Escherichia coli* strain (ATCC® 25922™) and 19 clinical multidrug-resistant strains isolated from the feces of pigs, dogs, calves, and goats (Supplementary Table S1).

### 2.2 Minimum inhibitory concentration determination

Broth microdilution was conducted using Mueller–Hinton broth (Hope Bio, Qingdao, China) with an inoculum of 1 × 10^6^ CFUs/ml^−1^. The plates were incubated at 37 °C for 16–20 h, and the MIC was defined as the lowest concentration with no visible bacterial growth. Since cefquinome is not included in the Clinical and Laboratory Standards Institute (CLSI) or the European Committee on Antimicrobial Susceptibility Testing (EUCAST) guidelines, clinical breakpoints for cefquinome were determined based on previous research (Vilaró et al., 2022). *Escherichia coli* ATCC® 25,922™ was used as the quality-control strain, and all tests were performed in triplicate.

### 2.3 Checkerboard assay

The combined effect of cefquinome and palmatine was evaluated using a checkerboard assay. Briefly, 100 μl of Mueller–Hinton broth was added to each well of a 96-well plate. Cefquinome and palmatine were serially diluted twofold along the x- and y-axes of the plate, respectively. An overnight bacterial culture was diluted 1:100 in Mueller–Hinton broth until the optical density at 600 nm (OD_600_) reached 0.1. The plates were then incubated at 37 °C for 1–20 h, after which the turbidity of each well was measured to assess bacterial growth.

The fractional inhibitory concentration index (FICI) for 20 isolates was measured in triplicate, and the FICI was calculated using the following formula:


$$\text{FICI}=\frac{\text{MICpalmatine}\;(\text{combo})}{\text{MICpalmatine}\;(\text{alone})}+\frac{\text{MICcefquinome}\;(\text{combo})}{\text{MICcefquinome}\;(\text{alone})}$$

The synergistic effect is defined as the effect observed when the FIC index value is ≤ 0.5 (Wachino et al., 2020).

### 2.4 Growth curve

The tested strains were incubated in LB broth (Land Bridge Technology, Beijing, China) at 37 °C until the OD_600_ reached 0.1. After dilution, the strains were evenly divided into conical bottles containing either no palmatine or varying concentrations of palmatine. The initial concentration of bacteria was 5 × 10^5^ CFU (Zhou et al., 2019).

### 2.5 Time-kill curves

Time-kill assays were used to evaluate the killing effects of cefquinome and palmatine against the tested strains, alone or in combination, by measuring the number of live bacteria at time points up to 24 h. The concentrations of cefquinome and palmatine in the culture medium of *E. coli* were 0.25 × MIC for the tested strains; the control medium did not contain these drugs. All samples were cultivated at 37 °C. After 0, 1, 3, 5, 7, and 24 h of incubation, 100 μl samples were taken, respectively, and diluted with sterile normal saline. The diluted samples were then inoculated on agar plates at 37 °C overnight, and the number of CFUs was calculated. Each assay was repeated in triplicate.

### 2.6 Drug-resistance development studies

Overnight bacterial cultures were inoculated into fresh Mueller–Hinton broth media containing either no drug, 0.25 × MIC cefquinome, or 0.25 × MIC cefquinome combined with 0.25 × MIC palmatine. After incubating at 37 °C for 24 h, the cultures were used to determine MIC values in triplicate. Meanwhile, each culture was diluted and transferred to fresh medium to establish the next generation, maintaining the same drug concentrations as in the previous cycle. This process was continued for 10 d.

### 2.7 Scanning electron microscope (SEM)

The tested strains were cultured in MH broth with 0.25 × MIC palmatine, 0.25 × MIC cefquinome, or 0.25 × MIC of the combined drugs for 10 h, respectively. Drug-free MH broth was used as a control. After 10 h, the bacteria were collected and washed with PBS. They were then fixed in 4% glutaraldehyde for 3 h, followed by dehydration in graded ethanol. Before scanning electron microscopy, the bacteria were subjected to critical-point drying in carbon dioxide and gold spraying.

### 2.8 Anti-biofilm activity assay

For the biofilm inhibition test, 96-well polystyrene plates were filled with 100 μl of an overnight-cultured bacterial suspension and 100 μl of medium containing either drug-free medium or 0.25 × MIC of single or combination drugs. The biofilm was then cultivated by incubating at 37 °C for 48 h. The bacterial precipitate was washed three times with sterile PBS to remove the floating bacteria. Then, 200 μl of methanol was added to each well for 15 min, and the precipitate was naturally air dried. Each well was stained with 200 μl of 1% crystal violet, incubated at room temperature for 5 min, and washed three times with sterile PBS to remove excess dye. After 10 min of incubation at room temperature, 200 μl of 95% ethanol was added, and the bound crystal violet was eluted. Biofilm formation was measured at OD_570_ nm. All tests were conducted three times, with MH broth as a negative control (Hou et al., 2022).

Confocal laser scanning microscopy (CLSM) was used to assess the inhibitory effects of different drugs on biofilms. Biofilms of the tested strains were cultivated in LB medium in 24-well, glass-bottomed microplates for 24 h at 37 °C under static conditions. The biofilms were then subjected to the drug treatments described above for 24 h, washed, and fixed with paraformaldehyde. Biofilm cells were stained with fluorescent dyes by adding 5 μM of SYTO® 9 green fluorescent nucleic acid stain (Invitrogen, Thermo Fisher Scientific; excitation at 488 nm and emission from 500 to 550 nm) and 5 μM of SYTO® 62 red fluorescent nucleic acid stain (Molecular Probes, Life Technologies; excitation at 652 nm and emission at 676 nm), prepared in 0.9% NaCl, incubated at room temperature for 15 min in the dark, and then washed twice with 0.9% NaCl (Tahrioui et al., 2019).

### 2.9 Bacteria staining and fluorescence assay

Multidrug-resistant *E. coli* E93 was clinically isolated and used in this fluorescence assay. Bacterial pretreatments for all fluorescence measurements were performed using similar protocols. Briefly, the tested bacteria were cultivated overnight at 37 °C, and the cultures were shaken at 200 rpm for 16 h. The tested bacteria were cultured in different medicated media, including drug-free, 0.25 × MIC cefquinome, 0.25 × MIC palmatine, or a combination. Then, the cultures were washed and suspended in PBS. The fluorescent dye was added when the suspension was adjusted to an OD_600_ of 0.5. After incubation at 37 °C for 30 min, the cultures were washed twice using PBS to remove excess unadsorbed fluorescent probes. Fluorescence intensity was measured. Several fluorescent probes were used in this assay as follows.

#### 2.9.1 Cell membrane integrity assay

The fluorescence intensity of 10 nM propidium iodide (PI) (Tsingke Biotech, Beijing, China) was measured at an excitation wavelength of 535 nm and an emission wavelength of 615 nm, either alone or in combination with cefquinome and palmatine.

#### 2.9.2 Outer membrane permeability assay

The fluorescence intensity of 10 μM 1-N-phenylnaphthylamine (NPN) (Macklin, Shanghai, China) was measured at an excitation wavelength of 350 nm and an emission wavelength of 420 nm, either alone or in combination with cefquinome and palmatine.

#### 2.9.3 Membrane depolarization assay

The fluorescence intensity of 0.5 μM 3,3-dipropylthiadicarbocyanine iodide DiSC_3_(5) (Macklin, Shanghai, China) was measured at an excitation wavelength of 622 nm and an emission wavelength of 670 nm, either alone or in combination with cefquinome and palmatine.

#### 2.9.4 Total ROS measurement

The fluorescence intensity of 0.5 μM 3,3-dipropylthiadicarbocyanine iodide DiSC_3_(5) (Macklin, Shanghai, China) was measured at an excitation wavelength of 622 nm and an emission wavelength of 670 nm, either alone or in combination with cefquinome and palmatine.

The levels of ROS in *E. coli* E93 were measured using 10 μM 2′,7′-dichlorodihydrofluorescein diacetate (DCFH-DA) (Beyotime, Shanghai, China). The fluorescence intensity was determined at an excitation wavelength of 488 nm and an emission wavelength of 525 nm.

### 2.10 Extracellular protein leakage assay

The bacterial suspensions cultured overnight were evenly mixed with sterile PBS containing 0.25 × MIC cefquinome, 0.25 × MIC palmatine, or both, respectively, and the same volume of drug-free PBS was used as a negative control. The supernatant obtained through centrifugation was incubated at 37 °C for 2 h. The supernatant was filtered through a 0.22-micron membrane, and the bacterial extracellular protein leakage was measured using a BCA protein concentration assay kit (Beyotime, Shanghai, China).

### 2.11 Transcriptome analysis

The tested *E. coli* E93 strain was grown in Mueller–Hinton broth to the early exponential phase and then treated with cefquinome (0.25 × MIC) alone or in combination with palmatine (0.25 × MIC) for 4 h. Each treatment was performed in triplicate. Total RNA was extracted using Trizol reagent (Takara, Tokyo, Japan), quantified using a NanoDrop™ spectrophotometer (Thermo Scientific, MA, USA), and sequenced using the Illumina HiSeq 2000 system (Genedenovo, Guangzhou, China).

mRNA library construction was conducted using the Illumina TruSeq RNA Sample Prep Kit according to the manufacturer’s protocol. Libraries were amplified by bridge PCR with the Illumina TruSeq PE Cluster Kit v3-cBot-HS on a cBot system and sequenced using the HiSeq2000 TruSeq SBS Kit v3-HS (200 cycles) with paired-end reads of 2 × 150 bp (PE150).

Raw sequencing reads were filtered and aligned to the *E. coli* E93 reference genome. Differentially expressed genes (DEGs) were identified using the FPKM (Fragments Per Kilobase of transcript per Million mapped reads) method, with significance thresholds of *p* ≤ 0.05 and fold change (FC) ≥ 2 (log_2_ FC ≥ 1 or log_2_ FC ≤ −1). DEGs were compared between the drug-free and cefquinome-treated groups and between the cefquinome-treated groups. Genes showing reversed expression patterns between these two comparisons were further analyzed.

### 2.12 RT-qPCR analysis

Sample processing in this experiment followed the same procedure as that used for transcriptome sequencing. *E. coli* E93 strains were grown to the early exponential phase, incubated, and then treated with cefquinome (0.25 × MIC) alone or in combination with palmatine (0.25 × MIC) for 4 h. Each treatment was performed in triplicate. Then, total RNA was extracted and quantified by its absorbance ratio (260 nm/280 nm). Reverse transcription was performed using the RT reagent kit with gDNA eraser (Dining, Beijing, China). Real-time quantitative PCR (RT-qPCR) was conducted using the SYBR Green qPCR kit (Dining, Beijing, China) on a Tianlong Technology system (Xi’an, China) with optimized primers (Supplementary Table S4). Gene expression levels were analyzed using the 2^-ΔΔCt^ method.

### 2.13 Swarming assay

Swarming Petri dishes (900 mm by 15 mm) contained 20 ml of medium supplemented with 1 mM MgSO_4_, 0.2% glucose, 0.5% casamino acids (Land Bridge Technology, Beijing, China), and 0.5% agar (Bru et al., 2019). The medium contained drugs, including 0.25 × MIC cefquinome or 0.25 × MIC combined drugs, and no drugs. The tested strain was cultured overnight (16 to 18 h) at 37 °C. Five microliters of culture were spotted in the center of the medium. Plates were incubated overnight at 37 °C, upright. The diameter of the swimming zone of the tested strain on each plate was measured, and each assay was repeated in triplicate.

### 2.14 Iron determination

The tested strain was cultured overnight from single colonies in LB medium until saturation. It was then inoculated into fresh LB broth containing either no drugs, 0.25 × MIC cefquinome, or 0.25 × MIC cefquinome combined with 0.25 × MIC palmatine, and incubated for 4 h. The bacterial cells were harvested by centrifugation and weighed. The total iron and ferrous ion contents in the bacterial pellets were measured using a Ferrous Ion Colorimetric Test Kit and a Total Iron Ion Colorimetric Assay Kit (Elabscience Biotechnology, Wuhan, China).

### 2.15 Taurine uptake inhibition test (a competitive inhibitor of taurine)

The synergistic effect of cefquinome and palmatine was assessed using a checkerboard assay with added taurine uptake inhibitors. β-Alanine is a competitive inhibitor of taurine uptake in *E. coli* (Jong et al., 2010). Moreover, 5 mM β-alanine was incorporated into Mueller–Hinton broth, and then the change in FICI (Fractional Inhibitory Concentration Index) of palmatine and cefquinome in the presence of the inhibitor was measured.

### 2.16 Animal studies

Female ICR mice aged 6–8 weeks were obtained from Chengdu Dossy Experimental Animals Co., Ltd. (Chengdu, China). The mice were kept at 18–22 °C with 40–60% humidity and acclimated for a week. The Sichuan Experimental Animal Management Committee issued the laboratory animal use license, number SCXK 2021–036. All procedures complied with Northwest A&F University’s laboratory animal management policies and the Guidelines for the Care and Use of Laboratory Animals (ISBN-10: 0–309–15,396-4).

### 2.17 Mouse systemic infection model

The ICR mice were challenged with a lethal dose of ETEC (1 × 10^9^ CFUs/ml^−1^, 300 μl) intraperitoneally to induce a systemic infection. The infected mice were randomly assigned to five groups (*n* = 10): cefquinome (5 mg/kg^−1^ of body weight), palmatine (10 mg/kg^−1^ of body weight), a combined drug group, a drug-free control (sterile saline), and a blank control. After 2 h of infection, different treatments were administered to each group of mice. Mouse survival was observed for up to 168 h post-infection.

### 2.18 Mouse model of acute diarrhea

The ICR mice were inoculated with ETEC (2.5 × 10^8^ CFUs/ml^−1^, 300 μl) to induce diarrhea. In our preliminary experiment, this dose caused diarrhea in all mice, and they all survived within 24 h. Female ICR mice infected with ETEC were randomly divided into four groups (*n* = 8). Before the pre-treatment injection, the mice were fasted for 12 h. At the start of the experiment, each mouse was placed in a separate cage covered with filter paper to record their fecal stains.

Mice in different groups were treated with the following treatments: cefquinome (5 mg/kg^−1^ of body weight, intraperitoneal injection), palmatine (10 mg/kg^−1^ of body weight, oral gavage), control treatment (intraperitoneal injection and oral gavage with an equal volume of sterile saline), and combination therapy administered 2 h after infection. The mice’s weights were recorded before and 24 h after infection. At 24 h after infection, blood samples were collected simultaneously from the orbit. The mice were anesthetized with Avertin (30 μl /g body weight) (DOWOBIO, Shanghai, China) and euthanized. Liver and intestine samples were then collected.

### 2.19 Evaluation of diarrhea symptoms in mice

Here, an evaluation criterion reflecting the severity of diarrhea in mice—loose stools recorded on filter paper—was used in this experiment. The loose stool incidence rate (LSIR) is calculated as the ratio of the number of loose stools to the total number of stools within an animal. The loose stool grade (LSG) is the degree of loose stools, classified into four grades according to the diameter of loose stools on the filter papers: Grade 1 (< 1 cm), Grade 2 (1–2 cm), Grade 3 (2–3 cm), and Grade 4 (>3 cm). The average loose stool grade (ALSG) is calculated as the ratio of the sum of LSGs of each loose stool to the total number of loose stools within an animal. The diarrhea index is calculated by multiplying LSIR by ALSG (Lu et al., 2020).

### 2.20 Determination of bacterial load in mouse tissue

The dilution plate count evaluated the bacterial load per unit mass of mouse tissue in each group. The collected mouse liver, jejunum, and ileum were weighed. Homogenized tissue and collected anticoagulant blood were diluted in sterilized normal saline containing Triton X-100 (0.05 mol L^−1^) in multiple dilutions and plated on an LB plate for overnight culture for colony counting. The bacterial content in the tissue was calculated according to the dilution ratio (Guan et al., 2019).

### 2.21 Statistical analysis

Statistical analysis was performed using GraphPad Prism 9 and SPSS 27.0. All data are presented as mean ± SD. For the *in vitro* studies, an unpaired t-test was used to calculate *p*-values for the RT-PCR assay, while one-way ANOVA was used for the other experiments. In the *in vivo* studies, n represented the number of animals per group, and statistical significance was determined by an unpaired t-test. Differences with a *p-value of* < 0.05 were considered significant. The number of asterisks indicates significance levels: *p < 0.05, **p < 0.01, ***p < 0.001.

## 3 Result

### 3.1 Synergistic activity of the cefquinome-palmatine combination

**Figure 1:**
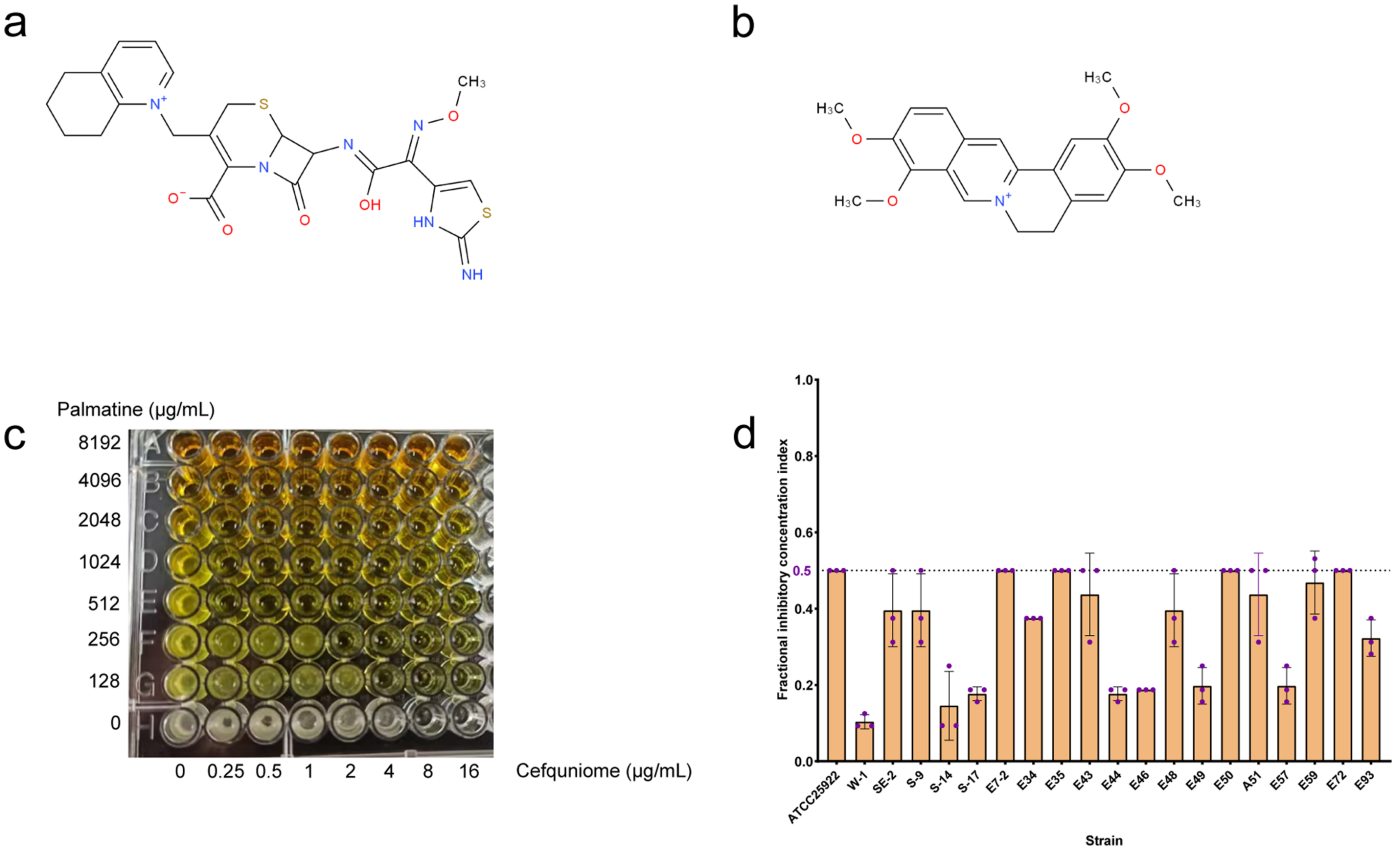
FIC index in tested bacterial strains. **(a)** Chemical structure of cefquinome. **(b)** Chemical structure of palmatine. **(c)** Synergistic effect of cefquinome and palmatine in the checkerboard assay. **(d)** FIC index from 20 tested strains of *E. coli.*

Cefquinome MICs of clinical isolates ranged from 2 to 1,024 μg/ml^−1^, and palmatine MICs ranged from 2,048 to 16,384 μg/ml^−1^ (Table 1). The MICs of cefquinome and palmatine for ATCC25922 were 0.08 ± 0.04 μg/ml^−1^ and 5461.33 ± 2364.83 μg/ml^−1^ (Table 1). Checkerboard assays revealed synergistic effects (FICI ≤ 0.5) among all 20 strains, with cefquinome MICs reduced by≥ 4-fold (Figure 1d). The mean FICI range was 0.10–0.50, demonstrating consistent potentiation across the tested strains.

**Table 1:** FICI results of MIC (Palmatine) and FICI (Palmatine × cefquinome) for 20 *E. coli* strains.

Strains	MIC (Alone)/μg ml^−1^		MIC (Combined)/μg ml^−1^		FICI
	Palmatine	Cefquinome	Palmatine	Cefquinome	
ATCC® 25,922™	5,500 ± 2,400	0.08 ± 0.04	1,400 ± 590	0.02 ± 0.01	0.50 ± 0.00
W-1	4,100 ± 0.00	8.0. ± 0.00	260 ± 0.00	0.42 ± 0.14	0.10 ± 0.02
SE-2	4,100 ± 0.00	510 ± 0.00	1,000 ± 0.00	75 ± 49	0.40 ± 0.10
S-9	8,200 ± 0.00	21 ± 9.2	2000 ± 0.00	2.7 ± 1.2	0.40 ± 0.10
S-14	4,100 ± 0.00	8.0 ± 0.00	260 ± 0.00	0.33 ± 0.14	0.10 ± 0.02
S-17	8,200 ± 0.00	8.0 ± 0.00	1,000 ± 0.00	0.42 ± 0.14	0.18 ± 0.02
E7-2	2000 ± 0.00	2.00 ± 0.00	510 ± 0.00	0.50 ± 0.00	0.50 ± 0.00
E34	2,700 ± 1,200	1,000 ± 0.00	850 ± 300	128 ± 0.00	0.38 ± 0.00
E35	4,100 ± 0.00	1,000 ± 0.00	1,000 ± 0.00	260 ± 0.00	0.50 ± 0.00
E43	2000 ± 0.00	21 ± 9.2	510 ± 0.00	3.3 ± 1.2	0.44 ± 0.11
E44	16,000 ± 0.00	4.0 ± 0.00	2000 ± 0.00	0.21 ± 0.07	0.18 ± 0.02
E46	16,000 ± 0.00	6.7 ± 2.3	2000 ± 0.00	0.42 ± 0.14	0.19 ± 0.00
E48	3,400 ± 1,200	32 ± 0.00	850 ± 300	4.7 ± 3.1	0.40 ± 0.10
E49	5,500 ± 2,400	16 ± 0.00	510 ± 0.00	1.2 ± 0.76	0.20 ± 0.05
E50	8,200 ± 0.00	1,000 ± 0.00	2000 ± 0.00	260 ± 0.00	0.50 ± 0.00
A51	3,400 ± 1,200	510 ± 0.00	850 ± 300	96 ± 55	0.44 ± 0.11
E57	4,100 ± 0.00	8.0 ± 0.00	510 ± 0.00	0.58 ± 0.38	0.20 ± 0.05
E59	4,100 ± 0.00	340 ± 150	1,400 ± 590	37 ± 24	0.47 ± 0.08
E72	4,100 ± 0.00	1,000 ± 0.00	1,000 ± 0.00	260 ± 0.00	0.50 ± 0.00
E93	2000 ± 0.00	1,000 ± 0.00	510 ± 0.00	75 ± 49	0.32 ± 0.05

The FICI data (Table 1), drug susceptibility results (Supplementary Table S2), and resistance gene profiles of the strains (Supplementary Figure S1) were comprehensively analyzed. The results revealed no direct correlation between the synergistic effect and specific drug-resistance genes or phenotypes. This conclusion is further validated by the consistent synergistic effect observed when combining these drugs with standard strains. Consequently, the synergistic interaction between palmatine and cefquinome exhibits a broad spectrum of activity.

### 3.2 Resistance suppression by palmatine

**Figure 2:**
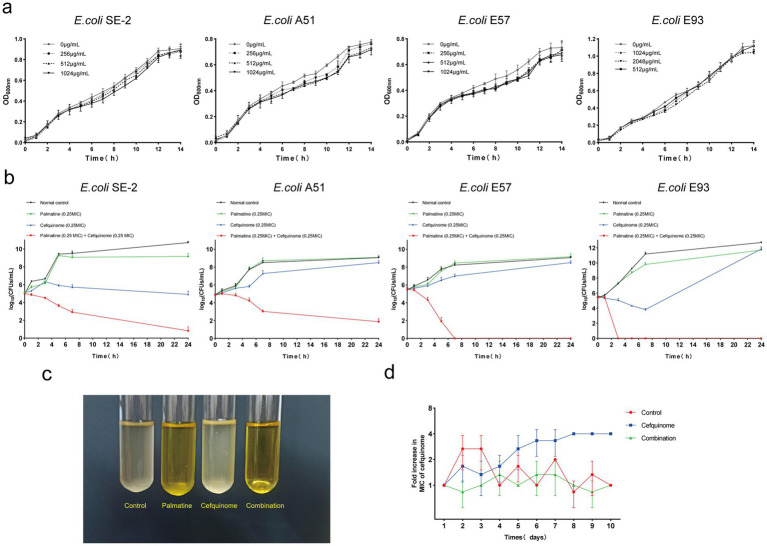
Palmatine improves the activity of cefquinome against *E.coli*. **(a)** Growth curve of *E. coli* at different subinhibitory concentrations (0.25 × MIC, 0.125 × MIC, and 0.0625 × MIC) of palmatine. **(b)** Time-dependent killing curve of *E. coli* treated with the combination of palmatine and cefquinome. *E. coli* was grown to the exponential phase and challenged with palmatine (0.25 × MIC) and cefquinome (0.25 × MIC) alone or in combination for 24 h. **(c)** The combination of palmatine (0.25 × MIC) and cefquinome (0.25 × MIC) can inhibit bacterial growth. **(d)** The addition of palmatine (0.25 × MIC) prevents the evolution of cefquinome resistance in *E. coli* E93 *in vitro*. Resistance is acquired during serial passaging in the presence of 0.25 × MIC levels of cefquinome (0.25 × MIC). All data are representative of three independent experiments and are shown as mean ± SD.

Growth curves confirmed that sub-MIC palmatine (0.25 × MIC) did not affect bacterial proliferation (Figure 2a). Time-kill assays showed that combination therapy (0.25 × MIC each) achieved > 4-log_10_ CFUs/ml^−1^ reduction within 24 h, surpassing monotherapy effects (Figures 2b,c). Serial passage experiments demonstrated that palmatine suppressed the development of cefquinome resistance: MICs increased fourfold in cefquinome-only groups but remained stable in combination cohorts (Figure 2d). These results suggest that palmatine treatment could prevent the growth of bacterial resistance.

### 3.3 Morphological and biofilm disruption

**Figure 3:**
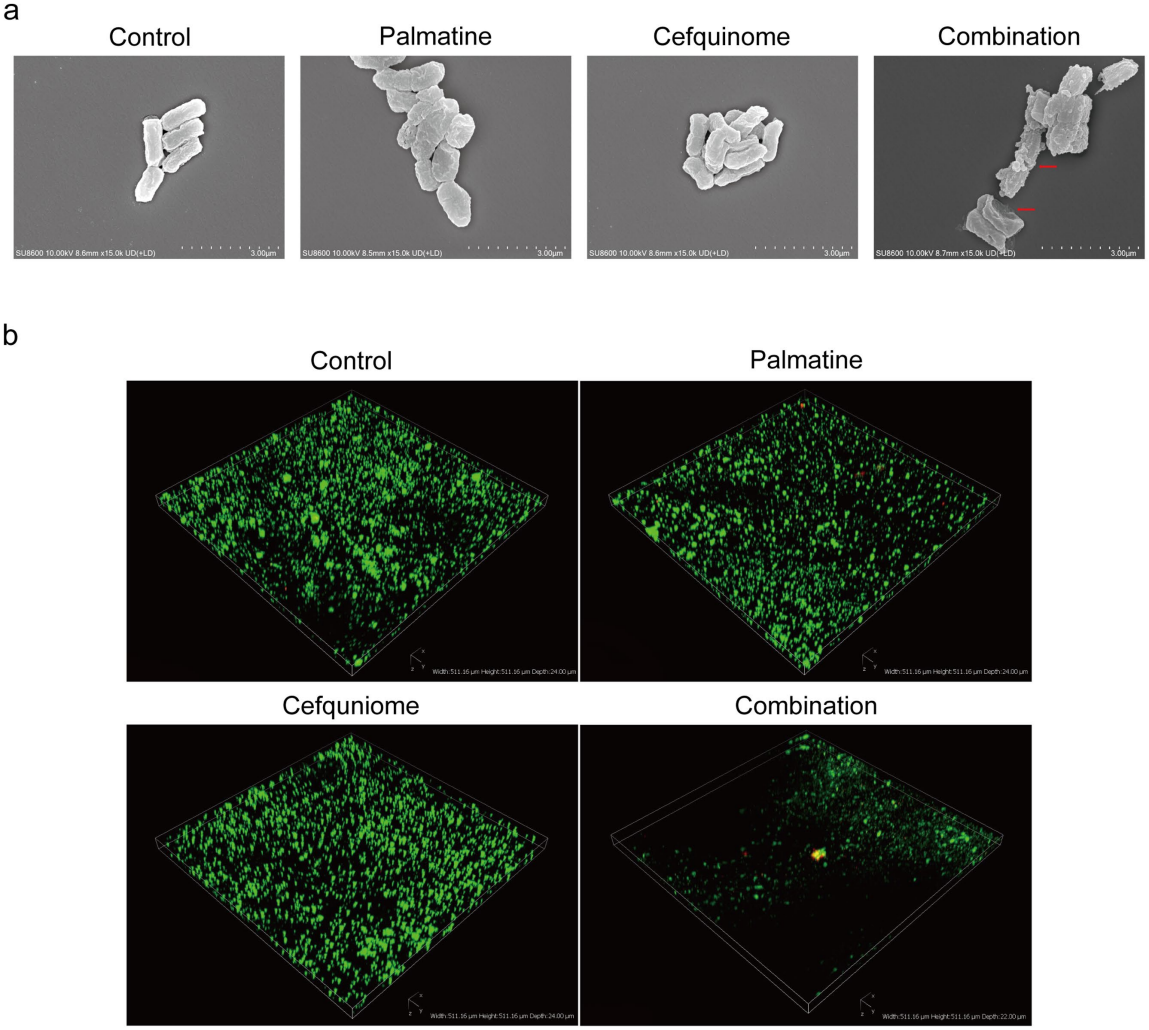
SEM and CLSI observation. **(a)** Palmatine enhances the damage caused by cefquinome to E.coli. Morphological changes in *E. coli* E93 treated with sub-MIC of palmatine, cefquinome, or their combination were visualized with SEM. Scale bars: 3.0 μm. **(b)** Three-dimensional reconstructions of 48-h *E.coli* biofilms were created using CLSM (200 × magnification). Red fluorescence represents dead bacteria, and green fluorescence represents live bacteria.

**Figure 4:**
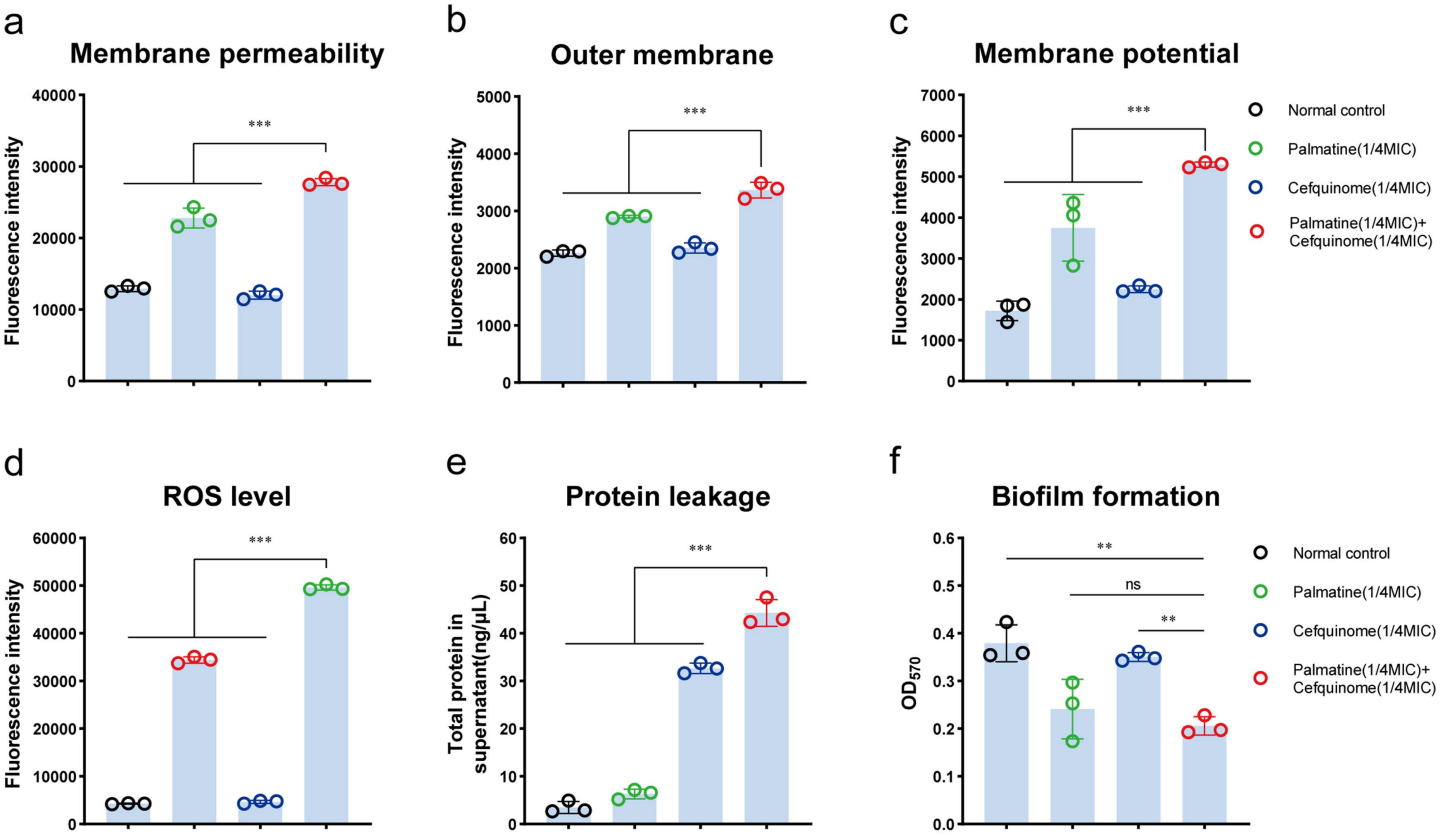
Palmatine enhances the destructive effect of cefquinome on *E.coli.* **(a)** Palmatine enhances the membrane permeability of cefquinome for propidium iodide (PI) in *E. coli.* **(b)** Palmatine permeabilizes the outer membrane and enhances cefquinome-induced outer membrane disruption. Permeability was evaluated by measuring the fluorescence intensity of 1-N-phenylnaphthylamine (NPN). **(c)** Palmatine dissipates membrane potential and drastically enhances cefquinome’s effects on it. Fluorescence dye DiSC_3_(5) was used to assess membrane potential changes induced by palmatine, cefquinome, or the combination. **(d)** The addition of palmatine significantly increases the production of ROS levels in *E.coli*; the lack of palmatine does not affect ROS levels. **(e)** The addition of palmatine further improves protein leakage in *E. coli* caused by cefquinome. **(f)** The addition of palmatine significantly inhibits *E. coli* biofilm formation; the lack of palmatine has no effect. All experiments were performed with biological replicates and are presented as mean ± SD. Concentrations of palmatine, cefquinome, or combination drugs were 0.25 MIC. One-way ANOVA among multiple groups was used to calculate *p*-values (*p < 0.05, **p < 0.01, ***p < 0.001).

Scanning electron microscopy (SEM) revealed severe membrane damage in combination-treated cells, including outer membrane perforations (Figure 3a, red arrows). CLSM analysis showed that the combination therapy reduced biofilm thickness and increased the number of cell deaths (Figure 3b). Crystal violet quantification confirmed that biofilm biomass decreased significantly after the combined treatment compared with antibiotics alone (*p* < 0.01) (Figure 4f).

### 3.4 Enhanced membrane damage and oxidative stress

We further verified that palmatine worsened the damage to bacterial cell membranes caused by cefquinome. To validate our hypothesis, we tested *E. coli* E93 with cefquinome (0.25 × MIC concentration) and found that the addition of palmatine could enhance the destructive effect of cefquinome on the bacterial outer membrane permeability (*p* < 0.001) (Figure 4a) and simultaneously cause dissipation of the cytoplasmic membrane potential (*p* < 0.001) (Figure 4b). Compared with the control group, palmatine monotherapy showed limited efficacy in reducing the intracellular protein leakage of *E. coli;* its combination with cefquinome significantly potentiated (*p* < 0.001) (Figure 4c).

In addition, intracellular ROS levels in *E. coli* increased with the addition of palmatine. The intracellular ROS concentration was also significantly higher in the combination group than in the monotherapy group, indicating that bacterial oxidative damage was intensified (*p* < 0.001) (Figure 4d).

### 3.5 Transcriptome analysis

**Figure 5:**
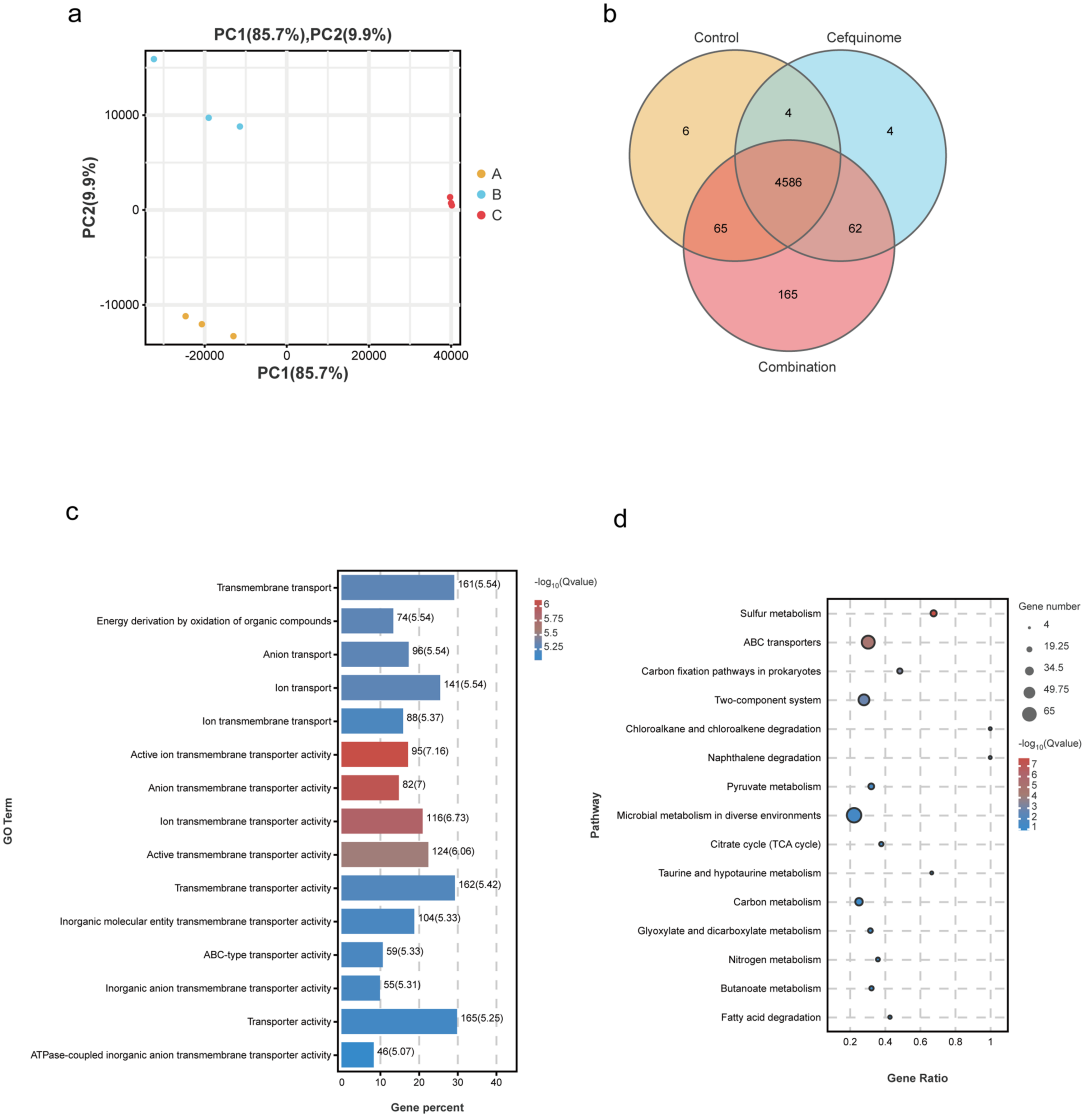
Transcriptomic analysis of *E. coli* E93 treated with no drug, palmatine, or the combination of cefquinome and palmatine. **(a)** Principal component analysis of different treatment groups of *E. coli* E93. **(b)** Venn diagrams showing the expressed genes in various treatment groups of *E. coli* E93. GO **(c)** and KEGG enrichment analysis **(d)** of the differentially expressed genes (DEGs) in *E. coli* E93 after exposure to no drug, cefquinome, or the combination of cefquinome and palmatine.

**Figure 6:**
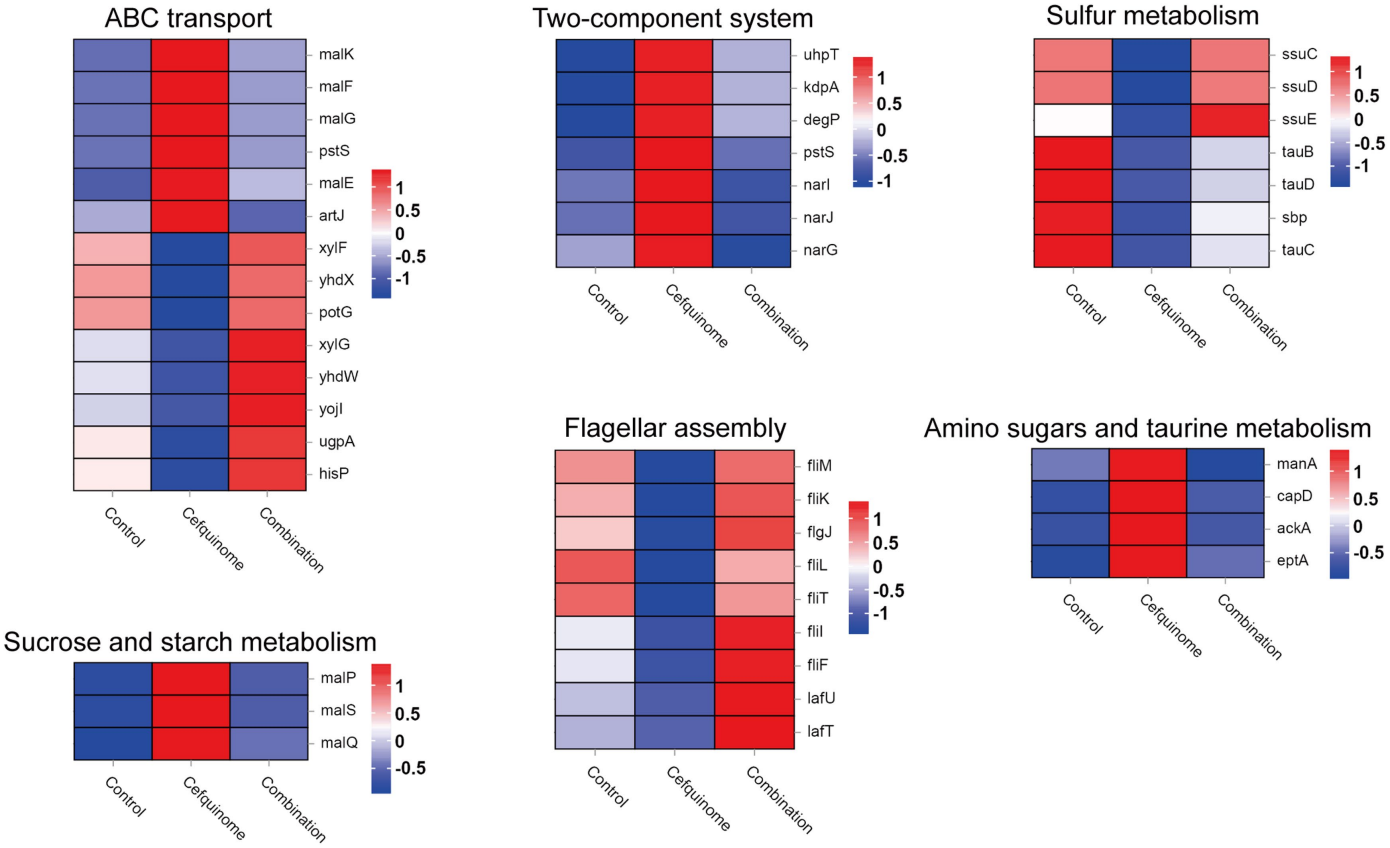
Transcriptomic differential gene. Filtered differentially expressed genes related to ABC transport, two-component systems, sulfur metabolism, sucrose and starch metabolism, flagellar assembly, amino sugar metabolism, and taurine metabolism. Three biological replicates were combined to produce the final data.

**Figure 7:**
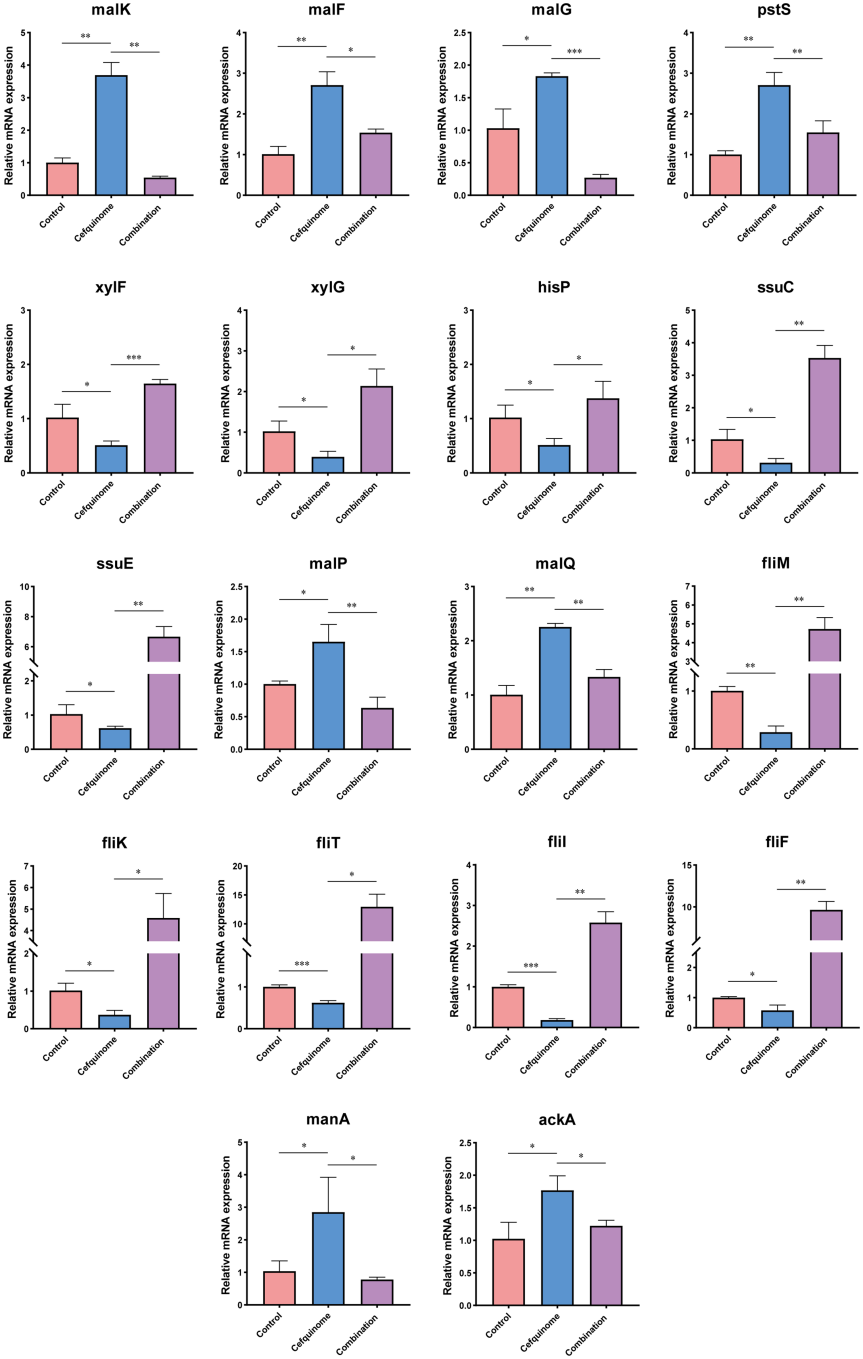
Validation of differential gene expression in transcriptomics. According to the results of transcriptome sequencing, relative expression of representative genes of *E. coli* E93 after treatment with cefquinome (0.25 × MIC) alone or in combination with palmatine (0.25 × MIC) for 4 h was determined by RT-PCR. Data are presented as mean ± SD, and an unpaired t-test was used to calculate *p*-values (*p < 0.05, **p < 0.01, ***p < 0.001).

We previously sequenced the whole genome of *E. coli* E93 using Nanopore sequencing to provide reference genomes for transcriptome sequencing (Supplementary Figure S2). Transcriptome analysis revealed 4,586 expressed genes across all treatment groups (Figure 5b). After further screening, 174 genes with reversed expression patterns under combination treatment compared to cefquinome alone were identified (Supplementary Table S3). GO and KEGG enrichment analyses highlighted significant changes in ABC transporters, flagellar assembly, two-component systems, and sulfur metabolism (Figures 5c,d). Notably, cefquinome-induced upregulation of genes involved in amino sugar and taurine metabolism was reversed by palmatine (Figure 6). RT-PCR validation confirmed the transcriptomic findings (Figure 7).

### 3.6 Functional validation of flagellar and iron metabolism

**Figure 8:**
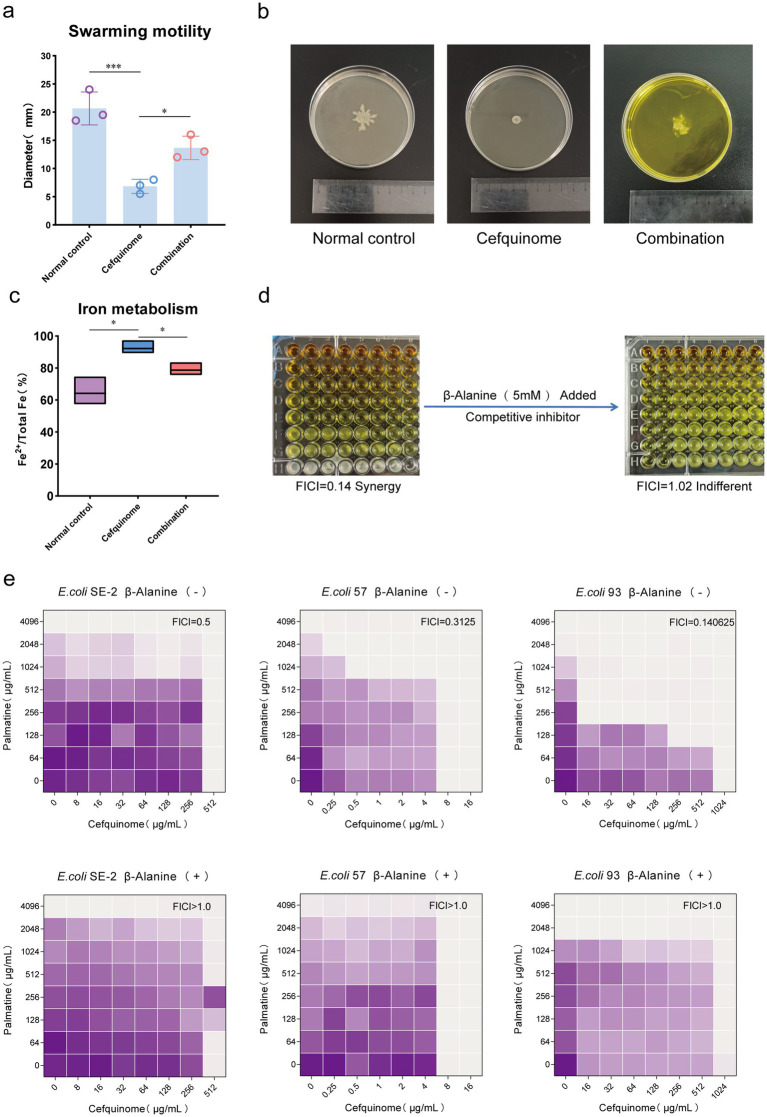
Phenotypic validation tests based on transcriptome sequencing. **(a,b)** The addition of palmatine restores the swarming motility of *E. coli* E93 under the stress of cefquinome. **(c)** The addition of palmatine restores the iron metabolism level (Fe^2+^ /total Fe) of *E. coli* E93 under the cefquinome stress. **(d)** The addition of taurine transport inhibitors (β-Alanine) eliminates the synergistic effect of palmatine and cefquinome. All experiments were performed with biological replicates and presented as mean ± SD. Concentrations of palmatine, cefquinome, or combination drugs were at 0.25 MIC. One-way ANOVA among multiple groups was used to calculate *p*-values (*p < 0.05, **p < 0.01, ***p < 0.001). **(e)** Checkerboard broth microdilution assays between palmatine and cefquinome against *E. coli* SE-2, *E. coli* E57, and *E. coli* E93 in the presence or absence of β-alanin. The mean OD at 600 nm of three biological replicates is presented.

Based on the differential change pathway identified by transcriptomic sequencing, we conducted a swarming assay and an iron metabolism experiment to confirm whether this pathway caused phenotypic changes. Bacterial flagellar movement controls swarming, and the two-component system regulates iron metabolism. Swarming assays showed that palmatine restored cefquinome-impaired motility, with swarming diameters increasing from 6.83 ± 1.26 mm to 13.67 ± 2.08 mm (*p* < 0.05) (Figures 8a,b). Iron quantification revealed that palmatine normalized the Fe^2+^/total Fe ratio, which was significantly elevated by cefquinome treatment (78.69% ± 4.29% vs. 92.21% ± 4.52%, *p* < 0.05) (Figure 8c).

### 3.7 Taurine metabolism mediates synergy

Our findings revealed a significant change in the synergistic effect when taurine uptake in the sulfur metabolic pathway of *Escherichia coli* was disrupted. The addition of β-alanine, a competitive inhibitor of taurine uptake, eliminated the synergistic effect of palmatine and cefquinome, shifting the FICI from 0.25 ± 0.03 to 1.12 ± 0.15 (*p* < 0.05) (Figures 8d,e). These results confirmed the critical role of taurine metabolism in the observed synergistic effect (Table 2).

**Table 2:** Fractional inhibitory concentration index (FICI) of tested strains before and after the addition of β-Alanine (a competitive inhibitor of taurine).

Isolates	FICI (β-Alanine−)	FICI (β-Alanine+)
E93	0.375, 0.3125, 0.140625	1.015625, 1.015625, 1.015625
E57	0.3125, 0.1875, 0.25	1.015625, 1.015625, 1.015625
SE-2	0.3125, 0.375, 0.5	1.0, 1.015625, 1.0

### 3.8 *In vivo* antibacterial efficacy of palmatine-cefquinome combination

**Figure 9:**
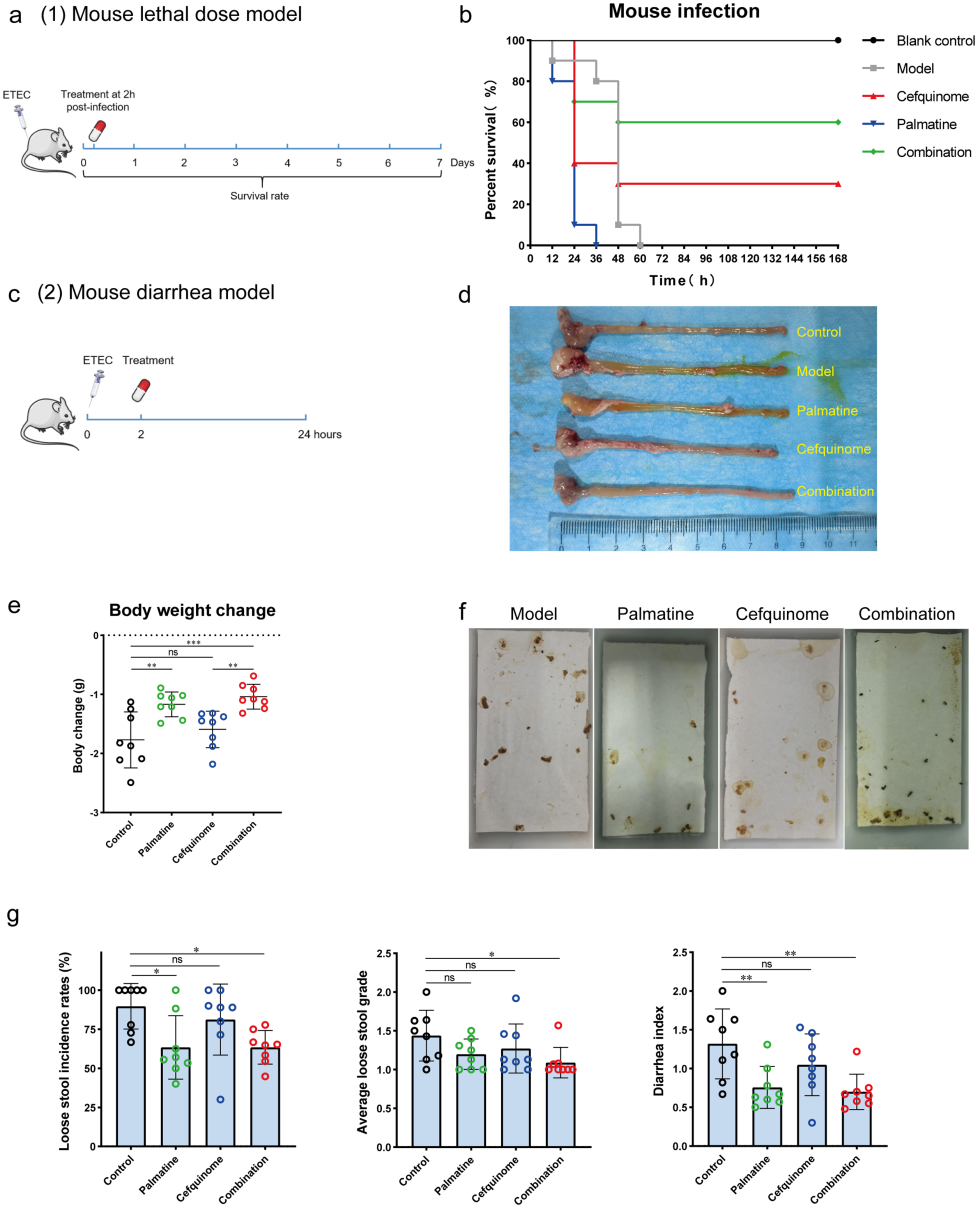
Palmatine restores cefquinome activity in two mouse infection models. **(a)** In the mouse lethal dose model, the mice were infected with a lethal dose (ETEC) of cefquinome-resistant *E. coli* E93 and treated with a single dose of cefquinome, palmatine, or their combination at 2 h post-infection and observed for 168 h. **(b)** A combination of cefquinome (5 mg kg^−1^) and palmatine (10 mg kg^−1^) improved the survival rate of mice (*n* = 10 per group) compared with other groups. **(c)** In the mouse diarrhea model, the mice were infected with cefquinome-resistant *E. coli* E57 at a certain dose (Supplementary Table S6; this dose caused diarrhea in all mice but did not cause acute death) and treated with a single dose of cefquinome, palmatine, or their combination at 2 h post-infection. Data were collected after 24 h. **(d)** The macroscopic pathological changes in mice 24 h post-infection. **(e)** Body weight changes in mice before and after 24 h of infection. **(f)** Pictures of loose stools on the filter paper for each group were used to assess the diarrhea situation. **(g)** Loose stool incidence rates, average loose stool grade, and diarrhea index were recorded to evaluate diarrhea status. All experiments were performed with biological replicates and are presented as mean ± SD. One-way ANOVA among multiple groups was used to calculate *p*-values (*p < 0.05, **p < 0.01, ***p < 0.001).

To evaluate the *in vivo* therapeutic potential of the palmatine-cefquinome combination, we established two murine models using *E. coli* 57 (ETEC): a systemic infection model (Figure 9a) and an ETEC diarrhea model (Figure 9c).

Systemic infection model: During the 7-day observation period, all mice in the untreated control and palmatine monotherapy groups succumbed to infection. In contrast, the combination of palmatine and cefquinome significantly improved survival rates from 30% (cefquinome alone) to 60% (Figure 9b).

ETEC diarrhea model: Mice in the combination group exhibited the least weight loss and differed significantly from those in the cefquinome monotherapy group (*p* < 0.001) within 24 h post-infection (Figure 9e). Histopathology (HE staining) supported reduced tissue damage in combo-treated mice (Supplementary Figure S3). The incidence of loose stools (LSIR) (*p* < 0.05), the mean loose stools grade (ALSG) (*p* < 0.05), and the diarrhea index (*p* < 0.01) were lowest among all groups and were significantly lower than in the control group. At the same time, there was no significant difference in the cefquinome monotherapy group (*p* > 0.05) (Figures 9f,g).

**Figure 10:**
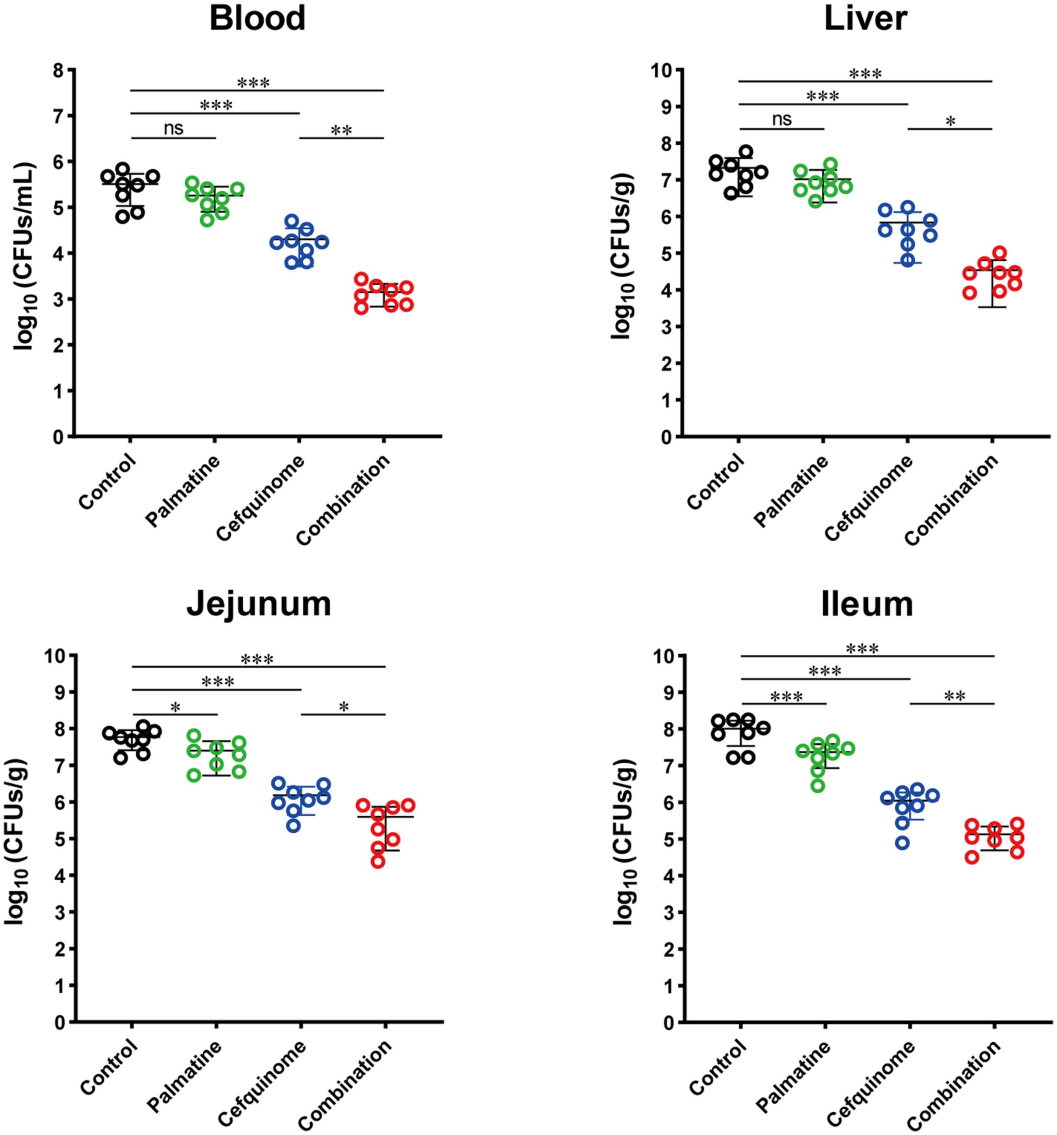
Palmatine restores cefquinome activity to lower bacterial load and tissue damage in the infected mice. At 24 h post-infection, mice infected with *E. coli* 57 were euthanized, and bacterial loads were determined in the blood, liver, jejunum, and ileum. All experiments were performed with biological replicates and presented as mean ± SD. One-way ANOVA among multiple groups was used to calculate *p*-values (*p < 0.05, **p < 0.01, ***p < 0.001).

Quantification of bacterial loads in key tissues demonstrated that the combination treatment significantly reduced colonization in the jejunum (*p* < 0.05), ileum (*p* < 0.01), blood (*p* < 0.01), and liver (*p* < 0.05) compared to the cefquinome monotherapy group (Figure 10). These findings collectively demonstrate that palmatine potentiates the antibacterial activity of cefquinome in vivo, effectively mitigating ETEC-induced systemic infection and diarrheal pathology while enhancing bacterial clearance.

## 4 Discussion

### 4.1 The emerging role of phytochemical adjuvants in combating antibiotic resistance

The global health crisis posed by multidrug-resistant Gram-negative bacteria demands urgent, innovative solutions (Ruegsegger et al., 2022; Tamma and Simner, 2018). With β-lactams losing efficacy against rapidly evolving resistance mechanisms (Kim et al., 2017; López-Jácome et al., 2022), the agricultural use of cephalosporins contributes to the dissemination of zoonotic resistance genes, particularly in veterinary cephalosporins such as the sole fourth-generation cefquinome. Palmatine’s ability to restore drug efficacy in animal-derived strains may disrupt this transmission chain. Adjuvant therapy emerges as a strategic countermeasure (Scientific Advisory Group on Antimicrobials of the Committee for Medicinal Products for Veterinary Use, 2009). This approach leverages existing antibiotics while reducing the pressure to develop resistance, which is a critical advantage given the stagnation in novel antibiotic discovery (Wright, 2016).

Our study identifies palmatine, a bioactive isoquinoline alkaloid from *Fibraurea recisa* Pierre, as a potent synergist for cefquinome against resistant *E. coli*. While previous studies have established palmatine’s inhibition of *Helicobacter pylori* and *Escherichia coli*, and its efflux pump inhibition against *Pseudomonas aeruginosa*, we reveal novel mechanisms underlying its synergistic effect with β-lactams (Aghayan et al., 2017; Chen et al., 2020; Rong et al., 2016; Wang P. et al., 2020). Crucially, this synergistic effect transcends specific resistance phenotypes or genotypes, showing consistent potentiation even in reference strains—a finding with broad therapeutic implications.

### 4.2 Mechanistic insights into synergistic action

Palmatine significantly enhanced cefquinome’s membrane-disruptive capacity at sub-inhibitory concentrations (0.25 × MIC), inducing 1.4-fold greater depolarization and a 42% increase in permeability. This membrane destabilization complements β-lactams’ inhibition of cell wall synthesis, creating a dual structural compromise. Furthermore, the combination generated 9.7-fold higher ROS levels than cefquinome monotherapy, exacerbating oxidative damage—a critical factor in bacterial clearance.

Biofilm analysis revealed that palmatine reduced biofilm formation by 36.34% at sub-MIC levels, whereas cefquinome alone showed no effect. The combination further increased biofilm-associated cell death by 9.33%, suggesting that palmatine disrupts this key resistance mechanism. Transcriptomic profiling revealed reversals in several vital pathways: cefquinome upregulated two-component systems and influenced the expression of ABC transporters—known resistance mediators (Azi et al., 2022; Liu et al., 2020)—while downregulating flagellar assembly and sulfur metabolism. Palmatine cotreatment reversed all these adaptations, normalizing nine flagellar genes and seven sulfur metabolism genes to baseline expression levels.

### 4.3 Resistance modulation and metabolic reversal

The observed trade-off between flagellar motility and antibiotic resistance supports the resource-allocation theory of microbial physiology. By forcing bacteria to divert energy toward flagellar assembly, palmatine disrupts the metabolic resources needed for efflux pump expression and biofilm matrix production (Lyu et al., 2021)—a vulnerability that can be exploited to reverse resistance.

The role of two-component systems (TCS) pathways as environmental sensors and resistance mediators was particularly evident (Azi et al., 2022; Diagne et al., 2022). Palmatine suppressed cefquinome-induced TCS upregulation, potentially disrupting bacterial stress adaptation. Studies have shown that sulfur metabolism is severely inhibited under β-lactam stress (Bie et al., 2023).

Palmatine effectively reverse-transcribes changes in this pathway, suggesting that metabolic resetting may be a new mechanism for regulating bacterial resistance. The disappearance of the synergistic effect was confirmed by functional inhibition of sulfur metabolism, thereby confirming the key role of this pathway. However, there are few studies on sulfur metabolism at present. To the best of our knowledge, this is the first time that restoring regulatory levels in sulfur metabolism has been associated with the enhanced antimicrobial effects of β-lactams against Gram-negative pathogens. In future research, we need to further investigate the in-depth mechanisms underlying sulfur metabolism regulation and bacterial resistance, providing theoretical support for preventing and controlling antibiotic resistance from a new perspective.

### 4.4 Innovative perspectives on resistance mechanisms

Our research reveals three significant findings: Palmatine synergistically enhances β-lactams’ membrane-disruptive activity by altering membrane dynamics. By forcing energy reallocation from resistance mechanisms to motility, the upregulation of flagellar genes (seven genes restored) breaks the trade-off that bacteria face when allocating energy in response to antibiotic stress—an evolutionary trap that increases antibiotic sensitivity. Under antibiotic stress, the recovery of sulfur metabolism disrupts the bacteria’s survival strategy and diminishes their ability to withstand it.

### 4.5 Therapeutic implications and future directions

According to this study, palmatine is a multi-target adjuvant that can reverse bacterial metabolic adaptations to antibiotic stress, interfere with biofilm defenses, and circumvent efflux pump resistance. A new research paradigm has emerged, recognizing that β-lactam resistance is linked to sulfur metabolism. Although earlier research concentrated on membrane proteins and efflux mechanisms (Benameur et al., 2019; Wang et al., 2021), our results show that metabolic pathways are valuable candidates for reversing resistance. The alterations in β-lactam resistance in bacteria will be investigated by expressing and knocking down sulfur metabolic pathway genes using molecular biology of sulfur. Future research should focus on confirming the impact of sulfur metabolism on antibacterial activity in Enterobacteriaceae *in vivo* and on investigating how iron homeostasis and two-component systems influence bacterial resistance to antibiotics.

## 5 Conclusion

Palmatine has been confirmed to have a new pharmacological effect: synergistically enhancing the antibacterial activity of the fourth-generation cephalosporin against *E. coli*. It could potentiate cefquinome through membrane disruption, ROS stimulation, and metabolic reset. This study moves phytochemical adjuvants from the identification of combination medications to the elucidation of their synergistic mechanisms. Given the lack of prior research in this field, uncovering the link between sulfur metabolism and bacterial resistance offers a new avenue for combating β-lactam resistance. Such logical adjuvant strategies provide a sustainable way to prolong the lifespan of antibiotics while addressing the resistance crisis.

## 6 Statement on use

Fibraureae Caulis, the dried vine stem of Fibraurea recisa, has a rich history of use in traditional Chinese medicine. It was first recorded in *The Compendium of Materia Medica* and in the Qing Dynasty’s *Lu Chuan Herbal Medicine*. It exhibits efficacy in purging heat and detoxifying, relieving constipation, promoting urination, and reducing swelling. It is used to treat constipation due to heat stagnation, dysentery, edema, abscesses, sores, carbuncles, and red eyes. Additionally, *Guangxi Chinese Herbal Medicine* records its efficacy in clearing heart fire and promoting urination, as well as its use in treating dysentery, acute gastroenteritis, acute tonsillitis, pharyngitis, conjunctivitis, tuberculosis, furuncles, scalds, and burns. For internal use, it is decocted in water with a daily dosage of 6–12 g. Palmatine is the main chemical component of Fibraureae Caulis and the main active ingredient. Palmatine’s effectiveness and safety have been fully recognized, and it is included in the *Chinese Pharmacopeia* (2020, Volume 1, page 433). Palmatine is used to treat infections, including gynecological inflammation, surgical infections, bacillary dysentery, and enteritis.

## Data Availability

The datasets presented in this study can be found in online repositories. The names of the repository/repositories and accession number(s) can be found at: https://www.ncbi.nlm.nih.gov/genbank/, CP112988, CP112989, CP112990, CP112991, CP112992, and CP112993.

## Supplementary material

SUPPLEMENTARY FIGURE S1 Drug resistance gene in tested bacterial strains. Drug resistance genes carried in 19 strains of *Escherichia coli*. Detected genes were marked with blue squares.

SUPPLEMENTARY FIGURE S2 Whole genome sequences of *E. coli* E93. Circular genome map of *E. coli* E93.

SUPPLEMENTARY FIGURE S3 Palmatine rescues cefquinome activity to reduce damage on tissue in infected mice. Histopathology of the jejunum tissues from *E. coli* 57 infected mice were assessed.
