# Supplementary figures and images for: Palmatine potentiates cefquinome efficacy against multidrug-resistant Escherichia coli via sulfur/taurine metabolism and oxidative stress modulation
Source: Front Microbiol. 2025 Nov 14;16:1644399. doi: 10.3389/fmicb.2025.1644399 (PMC12662166; doi:10.3389/fmicb.2025.1644399)

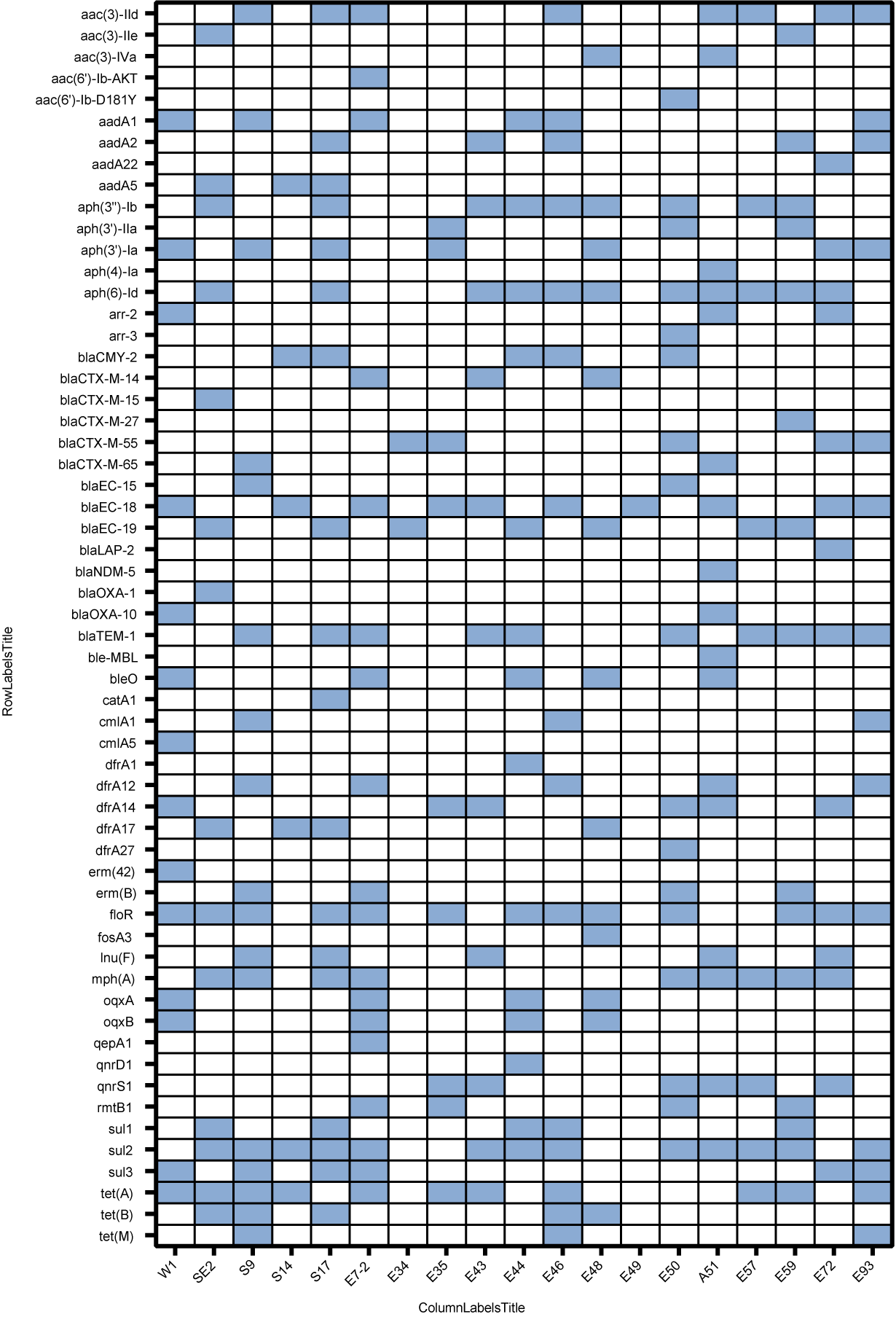

Supplement: SUPPLEMENTARY FIGURE S1 — Drug resistance gene in tested bacterial strains. Drug resistance genes carried in 19 strains of Escherichia coli. Detected genes were marked with blue squares. [file Image_1.tif]

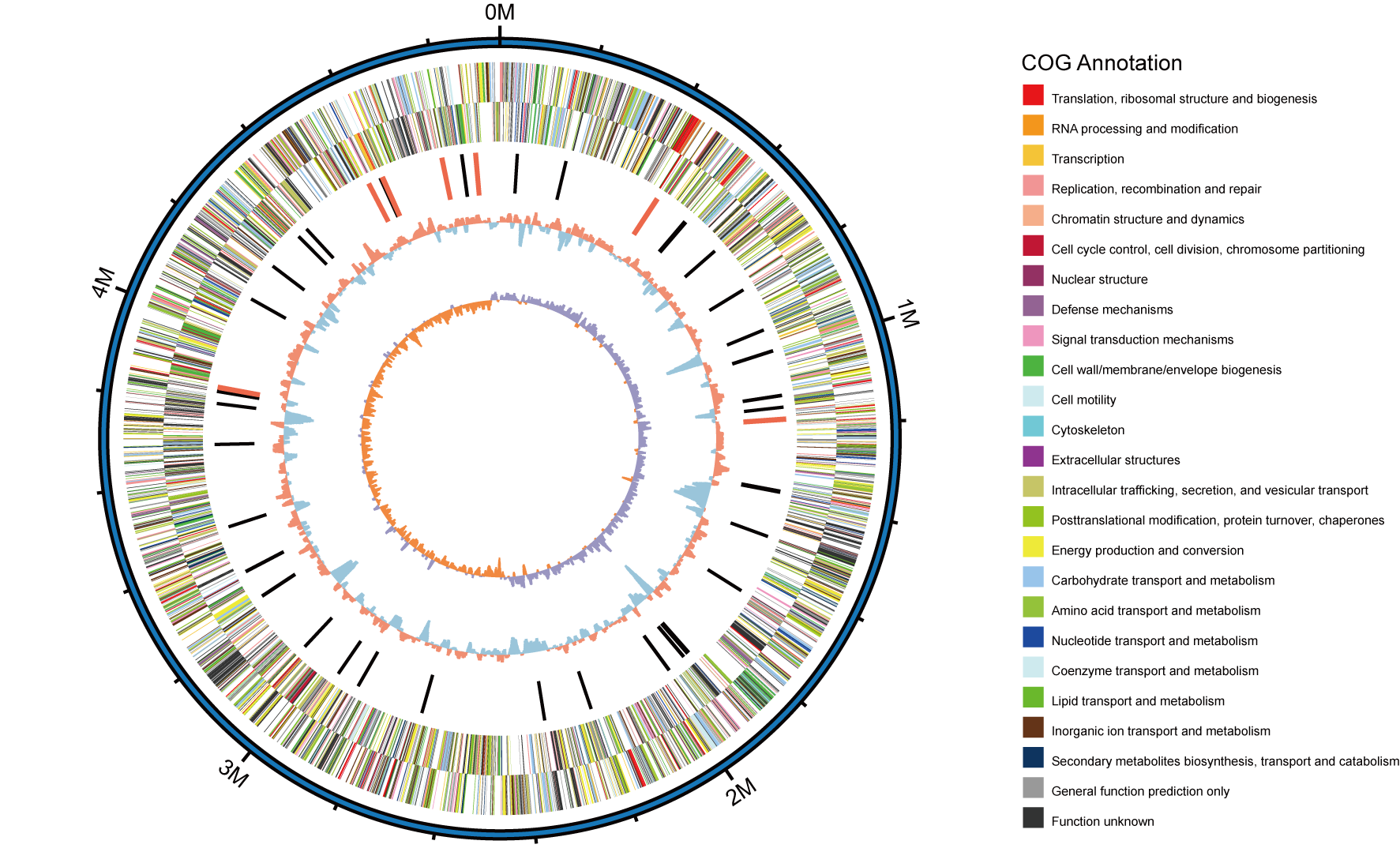

Supplement: SUPPLEMENTARY FIGURE S2 — Whole genome sequences of E. coli E93. Circular genome map of E. coli E93. [file Image_2.tif]

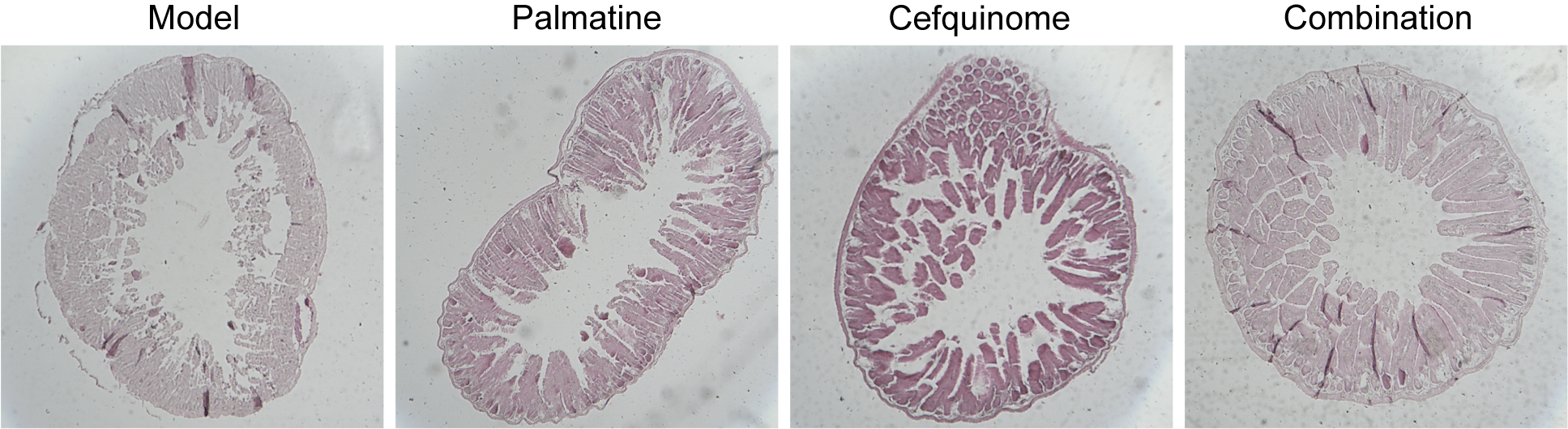

Supplement: SUPPLEMENTARY FIGURE S3 — Palmatine rescues cefquinome activity to reduce damage on tissue in infected mice. Histopathology of the jejunum tissues from E. coli 57 infected mice were assessed. [file Image_3.tif]
