# Supplementary material for: Palmatine potentiates cefquinome efficacy against multidrug-resistant Escherichia coli via sulfur/taurine metabolism and oxidative stress modulation
Source: Front Microbiol. 2025 Nov 14;16:1644399. doi: 10.3389/fmicb.2025.1644399 (PMC12662166; doi:10.3389/fmicb.2025.1644399)
Supplement: Supplementary file 6 [file Table_1.docx]

**Supplementary.**

**Table S1.** Strains of *E. coli* used in this study.

| **Strains** | **Source** |
| --- | --- |
| ATCC^®^ 25922^TM^ | In our laboratory |
| W-1 | Isolated from porcine |
| SE-2 | Isolated from porcine |
| S-9 | Isolated from porcine |
| S-14 | Isolated from porcine |
| S-17 | Isolated from porcine |
| E7-2 | Isolated from porcine |
| E34 | Isolated from cattle |
| E35 | Isolated from cattle |
| E43 | Isolated from cattle |
| E44 | Isolated from porcine |
| E46 | Isolated from porcine |
| E48 | Isolated from porcine |
| E49 | Isolated from canine |
| E50 | Isolated from canine |
| A51 | Isolated from capra hircas |
| E57 | Isolated from canine |
| E59 | Isolated from canine |
| E72 | Isolated from capra hircas |
| E93 | Isolated from porcine |

**Table S2.** Kirby-Bauer disk diffusion susceptibility test of *E. coli* strains.

| **Strains** | **GN** | **DOX** | **KAN** | **AK** | **CTX** | **TE** | **AMP** | **MEC** | **TOB** | **CIP** |
| --- | --- | --- | --- | --- | --- | --- | --- | --- | --- | --- |
| W-1 | I | R | R | R | R | R | R | S | S | R |
| SE-2 | R | R | R | R | R | R | R | S | S | R |
| S-9 | R | R | R | I | R | R | R | S | R | R |
| S-14 | R | R | I | R | R | R | R | S | S | R |
| S-17 | R | R | R | S | R | R | R | S | R | R |
| E7-2 | S | R | S | S | S | R | R | S | S | S |
| E34 | R | R | R | R | R | R | R | S | I | R |
| E35 | R | R | R | R | R | R | R | S | R | S |
| E43 | S | R | R | I | R | R | R | S | S | R |
| E44 | S | R | S | S | R | R | R | S | S | R |
| E46 | R | R | R | S | R | R | R | S | R | R |
| E48 | R | R | R | S | R | R | R | S | R | R |
| E49 | R | R | R | R | R | R | R | S | S | R |
| E50 | R | R | R | R | R | R | R | S | R | R |
| A51 | R | R | R | R | R | R | R | R | R | R |
| E57 | R | R | R | I | I | R | R | S | R | R |
| E59 | R | R | R | R | R | R | R | S | R | R |
| E72 | R | R | R | S | R | R | R | R | R | R |
| E93 | R | R | R | R | R | R | R | S | R | R |

GN: Gentamicin, DOX: Doxycycline, KAN:Kanamycin, AK: Amikacin, CTX: Cefotaxime, TE: Tetracycline, AMP: Ampicillin, MEC: Meropenem, TOB:Tobramycin, CIP: Ciprofloxacin.

S: suscepible, I : intermediate, R: resistant.

**Table S3.** Reversed change genes screened from Control VS Cefquinome

and Cefquinome VS Combination in this study.

| **Number** | **Genes** | **Control VS Cefquinome**  **log_2_ (FC)** | **Cefquinome VS Combination**  **log_2_ (FC)** |
| --- | --- | --- | --- |
| 1 | tauA | -5.065 | 3.542 |
| 2 | fliL | -4.439 | 4.115 |
| 3 | tauB | -4.333 | 2.853 |
| 4 | tauC | -3.770 | 2.529 |
| 5 | ssuE | -3.712 | 4.682 |
| 6 | yahL | -3.639 | 8.633 |
| 7 | sbp | -3.360 | 2.319 |
| 8 | arsR | -3.321 | 2.719 |
| 9 | rrrQ | -3.127 | 3.202 |
| 10 | ydfR | -3.099 | 3.264 |
| 11 | essD | -2.851 | 2.986 |
| 12 | yeaR | -2.807 | 6.354 |
| 13 | glcA | -2.528 | 1.447 |
| 14 | csgE | -2.522 | 5.489 |
| 15 | ssuA | -2.405 | 2.430 |
| 16 | tauD | -2.342 | 1.191 |
| 17 | ybdO | -2.261 | 5.749 |
| 18 | tfaQ | -2.261 | 2.967 |
| 19 | garP | -2.123 | 2.544 |
| 20 | ssuD | -2.116 | 2.104 |
| 21 | tetM | -1.996 | 2.052 |
| 22 | nanS | -1.994 | 3.634 |
| 23 | yicS | -1.993 | 2.674 |
| 24 | stfQ | -1.984 | 2.721 |
| 25 | rutE | -1.956 | 2.042 |
| 26 | nanM | -1.919 | 2.173 |
| 27 | lafU | -1.903 | 3.548 |
| 28 | dosC | -1.856 | 1.241 |
| 29 | dosP | -1.837 | 1.069 |
| 30 | phnJ | -1.826 | 3.812 |
| 31 | ygjJ | -1.820 | 4.805 |
| 32 | yjbT | -1.807 | 1.708 |
| 33 | ssuC | -1.784 | 1.786 |
| 34 | traX | -1.672 | 1.706 |
| 35 | mcbG | -1.576 | 3.079 |
| 36 | ugpA | -1.574 | 2.151 |
| 37 | ydjO | -1.573 | 4.910 |
| 38 | pdeC | -1.555 | 1.656 |
| 39 | wcaK | -1.547 | 1.888 |
| 40 | fliK | -1.538 | 1.786 |
| 41 | yiaU | -1.524 | 3.663 |
| 42 | csgD | -1.524 | 3.899 |
| 43 | bluF | -1.505 | 1.557 |
| 44 | rspA | -1.485 | 1.546 |
| 45 | abgA | -1.470 | 1.180 |
| 46 | livF | -1.463 | 4.814 |
| 47 | tnaB | -1.443 | 3.817 |
| 48 | yghR | -1.442 | 5.759 |
| 49 | yeiL | -1.439 | 1.897 |
| 50 | fiu | -1.419 | 2.330 |
| 51 | intQ | -1.407 | 3.867 |
| 52 | ltnD | -1.405 | 2.453 |
| 53 | yceJ | -1.394 | 2.555 |
| 54 | iraP | -1.386 | 1.482 |
| 55 | yphF | -1.374 | 3.305 |
| 56 | prpC | -1.370 | 5.459 |
| 57 | ydfU | -1.356 | 5.110 |
| 58 | flgJ | -1.350 | 1.723 |
| 59 | gadW | -1.340 | 1.442 |
| 60 | prpB | -1.318 | 5.624 |
| 61 | mcbR | -1.308 | 1.970 |
| 62 | mocA | -1.280 | 2.717 |
| 63 | ydhS | -1.279 | 1.220 |
| 64 | xylG | -1.275 | 2.251 |
| 65 | fliT | -1.275 | 1.163 |
| 66 | dmlA | -1.269 | 1.234 |
| 67 | yqiK | -1.260 | 5.160 |
| 68 | proV | -1.258 | 3.646 |
| 69 | garK | -1.246 | 2.207 |
| 70 | mqo | -1.239 | 1.521 |
| 71 | djlB | -1.221 | 3.604 |
| 72 | mcbF | -1.213 | 2.131 |
| 73 | yjgH | -1.198 | 1.516 |
| 74 | fliI | -1.196 | 2.020 |
| 75 | xylF | -1.196 | 1.419 |
| 76 | otnK | -1.190 | 1.763 |
| 77 | yfdK | -1.189 | 2.480 |
| 78 | fliF | -1.188 | 2.052 |
| 79 | ompF | -1.178 | 1.495 |
| 80 | yahG | -1.177 | 2.542 |
| 81 | potG | -1.172 | 1.280 |
| 82 | hprS | -1.169 | 2.488 |
| 83 | emrY | -1.161 | 4.189 |
| 84 | ybiU | -1.145 | 1.552 |
| 85 | yojI | -1.143 | 2.189 |
| 86 | mdtJ | -1.141 | 4.147 |
| 87 | hisP | -1.138 | 1.642 |
| 88 | ylaC | -1.138 | 2.096 |
| 89 | garR | -1.134 | 2.795 |
| 90 | ydcJ | -1.112 | 1.043 |
| 91 | ybiT | -1.096 | 1.005 |
| 92 | yhdW | -1.085 | 1.959 |
| 93 | kefB | -1.084 | 2.852 |
| 94 | yggP | -1.081 | 1.924 |
| 95 | lafT | -1.079 | 2.685 |
| 96 | yebB | -1.077 | 2.665 |
| 97 | yiaA | -1.069 | 3.008 |
| 98 | aceK | -1.061 | 2.375 |
| 99 | rhsB | -1.051 | 1.328 |
| 100 | hpaB | -1.043 | 2.121 |
| 101 | yciH | -1.033 | 1.400 |
| 102 | yidH | -1.033 | 1.175 |
| 103 | fliM | -1.029 | 1.108 |
| 104 | Bmul_0189 | -1.022 | 5.218 |
| 105 | ychO | -1.019 | 1.802 |
| 106 | ydcR | -1.018 | 1.044 |
| 107 | yegR | -1.018 | 1.894 |
| 108 | otnC | -1.015 | 1.306 |
| 109 | garL | -1.014 | 2.408 |
| 110 | yhdX | -1.006 | 1.105 |
| 111 | spy | 5.062 | -4.621 |
| 112 | malK | 4.889 | -2.647 |
| 113 | malE | 4.834 | -1.840 |
| 114 | malM | 4.484 | -2.081 |
| 115 | malF | 4.442 | -2.758 |
| 116 | lamB | 4.340 | -1.773 |
| 117 | yiaY | 4.261 | -1.238 |
| 118 | uhpT | 4.017 | -1.366 |
| 119 | malG | 3.986 | -2.605 |
| 120 | ycfJ | 3.896 | -1.566 |
| 121 | yhdV | 3.550 | -1.128 |
| 122 | malP | 3.419 | -2.371 |
| 123 | yncJ | 3.218 | -2.802 |
| 124 | yiaD | 3.186 | -1.658 |
| 125 | yaaX | 3.109 | -4.523 |
| 126 | ypeC | 3.048 | -2.111 |
| 127 | kdpA | 2.835 | -1.177 |
| 128 | cpxP | 2.800 | -1.987 |
| 129 | malQ | 2.742 | -1.622 |
| 130 | ecdD | 2.716 | -1.079 |
| 131 | hycC | 2.668 | -1.146 |
| 132 | malS | 2.578 | -1.956 |
| 133 | hycF | 2.533 | -1.221 |
| 134 | hycD | 2.520 | -1.270 |
| 135 | pstS | 2.493 | -1.934 |
| 136 | degP | 2.327 | -1.049 |
| 137 | yaiY | 2.324 | -1.714 |
| 138 | hycE | 2.317 | -1.126 |
| 139 | glnK | 2.119 | -1.403 |
| 140 | hycG | 2.015 | -1.305 |
| 141 | pspA | 1.896 | -2.079 |
| 142 | btsT | 1.839 | -4.241 |
| 143 | hisF3 | 1.833 | -1.204 |
| 144 | hokB | 1.767 | -6.421 |
| 145 | pta | 1.754 | -1.738 |
| 146 | mliC | 1.588 | -1.528 |
| 147 | eptA | 1.560 | -1.125 |
| 148 | yggR | 1.541 | -1.556 |
| 149 | nupC | 1.498 | -1.197 |
| 150 | ydfZ | 1.485 | -1.742 |
| 151 | narI | 1.470 | -1.938 |
| 152 | glpA | 1.442 | -1.396 |
| 153 | samA | 1.411 | -1.408 |
| 154 | ackA | 1.394 | -1.299 |
| 155 | ygdI | 1.321 | -4.327 |
| 156 | bhsA | 1.300 | -1.007 |
| 157 | hisH3 | 1.292 | -1.323 |
| 158 | ppiA | 1.288 | -1.277 |
| 159 | yuaY | 1.286 | -2.321 |
| 160 | artJ | 1.251 | -1.774 |
| 161 | narJ | 1.247 | -1.496 |
| 162 | eutM | 1.244 | -1.641 |
| 163 | yajG | 1.216 | -1.559 |
| 164 | mcjA | 1.202 | -6.715 |
| 165 | capD | 1.185 | -1.058 |
| 166 | nrfD | 1.167 | -1.164 |
| 167 | grxA | 1.158 | -1.557 |
| 168 | yccA | 1.134 | -1.237 |
| 169 | narG | 1.127 | -2.109 |
| 170 | ppiB | 1.092 | -1.432 |
| 171 | manA | 1.081 | -1.651 |
| 172 | hokD | 1.076 | -2.724 |
| 173 | pndA | 1.062 | -2.776 |
| 174 | lysU | 1.012 | -1.258 |

The differential genes with Log_2_(FC) >1.00 and *P*<0.05 were compared between the Control VS Cefquinome and Cefquinome VS Combination, and the change trend was reversed in them.

**Table S4.** Sequences of *E. coli* 16s rRNA and target gene primers used in PCR assays.

| **Gene** | **Sequence of Primer (5′~3′)** | **Size of Product/bp** | **Tm/°C** |
| --- | --- | --- | --- |
| 16s rRNA | F:ACTCCTACGGGAGGCAGCAG | 197 | 60.0 |
|  | R: ATTACCGCGGCTGCTGG |  |  |
| *fliF* | F:CTGCGTGCGAATCCGAAAAT | 85 | 60.0 |
|  | R:CTTTCGCCCACAGGATCAGT |  |  |
| fliI | F:TTGAGAACATCCTCGGTGCC | 149 | 60.0 |
|  | R:CAGCAACACATGCTGACCAC |  |  |
| fliK | F:GTTAACCACCGCTCAGACGA | 97 | 60.0 |
|  | R:TGCGGTGACGTCTTCATTCA |  |  |
| fliM | F: ATATCACCACCTCGCCGAAC | 108 | 60.0 |
|  | R:GCTCGATCATGCTGAATGGC |  |  |
| fliT | F:CGAAACTGGTCGGTCAGTCA | 94 | 60.0 |
|  | R:AAGAGGTTATCCTGCGGAGC |  |  |
| malK | F:ATTTGCTGGATCGCAAACCG | 94 | 60.0 |
|  | R:AAAAATACGCTTGGCTCGGC |  |  |
| *ackA* | F:TATGTAACCCAGGAAGCGGC | 131 | 60.0 |
|  | R:CCCATAGAGGTGTCAACGCA |  |  |
| malF | F:AAGCTAAGCCCCGGTTACAC | 99 | 60.0 |
|  | R:GACGAAAATGGCGAGGAACG |  |  |
| malG | F:GCTGCGTCAGGGAAACTTTG | 148 | 60.0 |
|  | R:GTTCCACAGCCACAGCAGTA |  |  |
| *malP* | F:ACGTCGCTGGATCAAACAGT | 144 | 60.0 |
|  | R:TCGCGATATTGCTGACGGAA |  |  |
| malQ | F:TGACCACCGAAGAAGGAACG | 104 | 60.0 |
|  | R: GGTGAGTGTCAGCGTGTGAT |  |  |
| pstS | F:ACTGGCTCAGGAAGGTCTGT | 105 | 60.0 |
|  | R:TTACCATCCAGCACCAGTTCG |  |  |
| ssuC | F:GGCTGTCGACGCGTATTTT | 133 | 60.0 |
|  | R:CCGCCAATTGAAAAGCCAA |  |  |
| ssuE | F:GCAAGGCAAAGTGGTGCTAC | 107 | 60.0 |
|  | R:TCTCCTGAGCTTTCAGTGCG |  |  |
| *xylF* | F:AAGCGCGCAAGGTTTATCAG | 135 | 60.0 |
|  | R:GCGGCAGTATTTGCCAACAA |  |  |
| *xylG* | F:CATCAGGAATTGGCCCTGGT | 122 | 60.0 |
|  | R:AGCAGCTTCTGACAGCGTAG |  |  |
| hisP | F: GGGGAAATATCCGGTGCATCT | 140 | 60.0 |
|  | R:GCAACACTTCGCCTACCAGT |  |  |
| manA | F:AAGCAGTTCACGAGTGCAGA | 150 | 60.0 |
|  | R:GGCTGTGCTGCGCATAATAC |  |  |

**Table S5.** Determination of median lethal dose (LD_50_) in ETEC infected mice.

| **Concentration**  **(CFUs mL^-1^)** | **Volume**  **(μL)** | **Number of mice** | **Number of deaths (72h )** | **72 h-mortality rate**  **(%)** |
| --- | --- | --- | --- | --- |
| 1 × 10^10^ | 300 | 6 | 6 | 100% |
| 1 × 10^9^ | 300 | 6 | 6 | 100% |
| 1 × 10^8^ | 300 | 6 | 0 | 0% |
| 1 × 10^7^ | 300 | 6 | 0 | 0% |

The median lethal dose was calculated based on Karber: LD_50_=3.16×10^8^ CFUs mL^-1^.

**Table S6.** Establishment of diarrhea mouse model.

| **Concentration**  **(CFUs mL^-1^)** | **Volume**  **(μL)** | **Number of mice** | **Number of diarrhea** | **Diarrhea rate (%)** | **24h-Mortality rate (%)** | **48h-Mortality rate (%)** | **72h-Mortality rate (%)** |
| --- | --- | --- | --- | --- | --- | --- | --- |
| 5 × 10^8^ | 300 | 6 | 4 | 66.67% | 16.67% | 33.33% | 83.33% |
| 2.5 × 10^8^ | 300 | 6 | 6 | 100% | 0 | 0 | 33.33% |
| 1 × 10^8^ | 300 | 6 | 3 | 50% | 0 | 0 | 0 |
| 5 × 10^7^ | 300 | 6 | 1 | 16.67% | 0 | 0 | 0 |

Mice injected with 300 μL of the bacteria at the concentration of 2.5 × 10^8^ CFUs mL^-1^ all developed diarrhea and were able to survive within 48 hours.
