# Supplementary material for: Palmatine potentiates cefquinome efficacy against multidrug-resistant Escherichia coli via sulfur/taurine metabolism and oxidative stress modulation
Source: Front Microbiol. 2025 Nov 14;16:1644399. doi: 10.3389/fmicb.2025.1644399 (PMC12662166; doi:10.3389/fmicb.2025.1644399)
Supplement: Supplementary file 5 [file Data_Sheet_1.pdf]

#FILE	./34ZYN.	./43ZYN.	./72ZYN.	./93ZYN.	./A51ZYN.	./S-9ZYN.	./W-1ZYN.	./ZYN35.	./ZYN44.	./ZYN46.	./ZYN48.	./ZYN49.	./ZYN5-1	./ZYN5-1	./ZYN50.	./ZYN57.	./ZYN59.	./ZYN7-2.	./ZYNSE-
NUM_FOUND	2	11	15	14	17	17	14	9	14	15	13	1	6	18	17	9	15	18	14
aac(3)-IId	.	.	100	100	100	100	.	.	.	100	.	.	.	100	.	100	.	100	.
aac(3)-IIe	.	.	.	.	.	.	.	.	.	.	.	.	.	.	.	.	100	.	100
aac(3)-IVa	.	.	.	.	100	.	.	.	.	.	100	.	.	.	.	.	.	.	.
aac(6')-Ib-AKT	.	.	.	.	.	.	.	.	.	.	.	.	.	.	.	.	.	100	.
aac(6')-Ib-D181Y	.	.	.	.	.	.	.	.	.	.	.	.	.	.	100	.	.	.	.
aadA1	.	.	.	100	.	100	82.58	.	100	100	.	.	.	.	.	.	.	100	.
aadA2	.	100	.	100	.	.	.	.	.	100	.	.	.	100	.	.	100	.	.
aadA22	.	.	100	.	.	.	.	.	.	.	.	.	.	.	.	.	.	.	.
aadA5	.	.	.	.	.	.	.	.	.	.	.	.	100	100	.	.	.	.	100
aph(3'')-Ib	.	99.88	.	.	.	.	.	.	99.88	99.88	99.88	.	.	99.88	96.52	99.88	99.88	.	99.88
aph(3')-IIa	.	.	.	.	.	.	.	100	.	.	.	.	.	.	100	.	100	.	.
aph(3')-Ia	.	.	100	100	.	100	100	100	.	.	100	.	.	100	.	.	.	.	.
aph(4)-Ia	.	.	.	.	100	.	.	.	.	.	.	.	.	.	.	.	.	.	.
aph(6)-Id	.	100	100	.	85.07	.	.	.	100	100	100	.	.	100	100	100	100	.	100
arr-2	.	.	100	.	100	.	100	.	.	.	.	.	.	.	.	.	.	.	.
arr-3	.	.	.	.	.	.	.	.	.	.	.	.	.	.	100	.	.	.	.
blaCMY-2	.	.	.	.	.	.	.	.	100	100	.	.	100	100	100	.	.	.	.
blaCTX-M-14	.	100	.	.	.	.	.	.	.	.	100	.	.	.	.	.	.	100	.
blaCTX-M-15	.	.	.	.	.	.	.	.	.	.	.	.	.	.	.	.	.	.	100
blaCTX-M-27	.	.	.	.	.	.	.	.	.	.	.	.	.	.	.	.	100	.	.
blaCTX-M-55	100	.	100	100	.	.	.	100	.	.	.	.	.	.	100	.	.	.	.
blaCTX-M-65	.	.	.	.	100	100	.	.	.	.	.	.	.	.	.	.	.	.	.
blaEC-15	.	.	.	.	.	100	.	.	.	.	.	.	.	.	100	.	.	.	.
blaEC-18	.	100	100	100	100	.	100	100	.	100	.	100	100	.	.	.	.	100	.
blaEC-19	100	.	.	.	.	.	.	.	100	.	100	.	.	100	.	100	100	.	100
blaLAP-2	.	.	100	.	.	.	.	.	.	.	.	.	.	.	.	.	.	.	.
blaNDM-5	.	.	.	.	100	.	.	.	.	.	.	.	.	.	.	.	.	.	.
blaOXA-1	.	.	.	.	.	.	.	.	.	.	.	.	.	.	.	.	.	.	100
blaOXA-10	.	.	.	.	100	.	100	.	.	.	.	.	.	.	.	.	.	.	.
blaTEM-1	.	100	100	100	.	100	.	.	100	.	.	.	.	100	86.06	100	100	100	.
ble-MBL	.	.	.	.	100	.	.	.	.	.	.	.	.	.	.	.	.	.	.
bleO	.	.	.	.	96.79	.	96.79	.	96.79	.	89.14	.	.	.	.	.	.	96.79	.
catA1	.	.	.	.	.	.	.	.	.	.	.	.	.	100	.	.	.	.	.
cmlA1	.	.	.	100	.	100	.	.	.	100	.	.	.	.	.	.	.	.	.
cmlA5	.	.	.	.	.	.	100	.	.	.	.	.	.	.	.	.	.	.	.
dfrA1	.	.	.	.	.	.	.	.	100	.	.	.	.	.	.	.	.	.	.
dfrA12	.	.	.	100	100	100	.	.	.	100	.	.	.	.	.	.	.	100	.
dfrA14	.	100	100	.	100	.	100	100	.	.	.	.	.	.	100	.	.	.	.
dfrA17	.	.	.	.	.	.	.	.	.	.	100	.	100	100	.	.	.	.	100
dfrA27	.	.	.	.	.	.	.	.	.	.	.	.	.	.	100	.	.	.	.
erm(42)	.	.	.	.	.	.	100	.	.	.	.	.	.	.	.	.	.	.	.
erm(B)	.	.	.	.	.	100	.	.	.	.	.	.	.	.	100	.	100	100	.
floR	.	.	100	100	.	100	100	100	100	100	100	.	.	100	100	.	100	100	100
fosA3	.	.	.	.	.	.	.	.	.	.	100	.	.	.	.	.	.	.	.
lnu(F)	.	92.58	100	.	100	100	.	.	.	.	.	.	.	92.58	.	.	.	.	.
mph(A)	.	.	100	.	100	100	.	.	.	.	.	.	.	100	100	100	100	100	100
oqxA	.	.	.	.	.	.	100	.	100	.	100	.	.	.	.	.	.	100	.
oqxB	.	.	.	.	.	.	100	.	100	.	100	.	.	.	.	.	.	100	.
qepA1	.	.	.	.	.	.	.	.	.	.	.	.	.	.	.	.	.	100	.
qnrD1	.	.	.	.	.	.	.	.	100	.	.	.	.	.	.	.	.	.	.
qnrS1	.	100	100	.	100	.	.	100	.	.	.	.	.	.	100	100	.	.	.
rmtB1	.	.	.	.	.	.	.	100	.	.	.	.	.	.	100	.	100	100	.
sul1	.	.	.	.	.	.	.	.	100	100	.	.	.	100	.	.	100	.	100
sul2	.	100	.	100	100	100	.	.	100	100	.	.	100	100	100	100	100	100	100
sul3	.	.	100	100	.	100	100	.	.	.	.	.	.	100	.	.	.	100	.
tet(A)	.	100	.	100	.	100	100	100	.	100	.	.	100	.	.	100	100	100	100
tet(B)	.	.	.	.	.	100	.	.	.	100	100	.	.	100	.	.	.	.	100
tet(M)	.	.	.	100	.	100	.	.	.	100	.	.	.	.	.	.	.	.	.
